# Distinct effects of supplementation with resistant starch and polydextrose on plasma and faecal bile acid profile and associations with gut microbiota: a randomised, controlled intervention in healthy participants

**DOI:** 10.1007/s00394-026-04060-1

**Published:** 2026-07-23

**Authors:** Jiemin Fan, Gwenaelle le Gall, Fiona C. Malcomson, Panayiotis Louca, Lauren Beck, Andrew Nelson, Naomi D. Willis, Iain McCallum, Long Xie, Arthur C. Ouwehand, Julian D. Stowell, Seamus B. Kelly, Michael Bradburn, Nigel J. Belshaw, Ian T. Johnson, Christopher J. Stewart, Michael Müller, Bernard M. Corfe, John C. Mathers

**Affiliations:** 1 Human Nutrition and Exercise Research Centre, Centre for Healthier Lives, Population Health Sciences Institute, Newcastle University, Newcastle upon Tyne, NE2 4HH UK; 2 Centre for Cancer, Population Health Sciences Institute, Newcastle University, Newcastle upon Tyne, NE2 4HH UK; 3 Faculty of Medicine and Health Sciences, Norwich Medical School, University of East Anglia, Norwich, NR4 7TJ UK; 4 Translational and Clinical Research Institute, Newcastle University, Newcastle upon Tyne, NE2 4HH UK; 5 Department of Applied Science, Northumbria University, Newcastle upon Tyne, NE1 8ST UK; 6 Northumbria Healthcare NHS Foundation Trust, North Tyneside General Hospital, Rake Lane, North Shields, NE29 8NH UK; 7 IFF Health, 02460 Kantvik, Finland; 8 Sabri Ülker Foundation, Istanbul, Turkey; 9 Northumbria Healthcare National Health Service Foundation Trust, Ashington, NE63 9JJ UK; 10 Quadram Institute, Norwich Research Park, Norwich, Norfolk, NR4 7UQ UK

**Keywords:** Bile acid, Resistant starch, Polydextrose, Gut microbiota

## Abstract

**Purpose:**

Dietary fibre may influence bile acid (BA) metabolism via interactions with gut microbiota. We hypothesised that dietary fibres with distinct fermentative properties, resistant starch (RS) and polydextrose (PD), would differentially alter BA profiles in plasma and faeces through gut microbiota-mediated mechanisms.

**Methods:**

BA profiles were analysed by ultra-performance liquid chromatography mass spectrometry in plasma (*n* = 74) and faeces (*n* = 50) from a double-blind, randomised, placebo-controlled 2 × 2 factorial trial. Healthy participants consumed 23 g/day Hi-maize^®^260 (type 2 RS) and/or 12 g/day Litesse^®^Ultra™ (PD) for 50 days. The intervention effects of RS and PD on BA profile were investigated using general linear models and beta regression models. Genus abundances derived from 16 S rRNA gene sequencing were used to investigate fibre-specific microbial correlations with BA profiles.

**Results:**

Supplementation with RS, but not PD, increased a range of conjugated BAs and deoxycholic acid (FDR < 0.05). Concentrations of taurochenodeoxycholic acid (FDR = 0.027) and taurine conjugated BAs (FDR = 0.049) in plasma correlated positively with *Akkermansia* abundance in response to RS. Although neither RS nor PD altered BA concentrations in faeces, RS decreased (*p* = 0.032) and PD increased (*p* = 0.012) faecal proportions of primary BAs. PD reduced secondary BA transformation ratios (*p* < 0.05), along with shifts in related microbial associations. There were negative correlations between plasma primary conjugated BAs and faecal secondary BAs in response to RS specifically (*p* < 0.05).

**Conclusion:**

RS increased plasma BAs, particularly conjugated BAs, whereas PD reduced faecal secondary BA transformation. The distinct impacts of RS and PD on BA profiles and fibre-specific microbial associations may underlie their differential metabolic effects.

*Trail registration* The DISC Study was registered with https://clinicaltrials.gov/ (Identifier NCT01214681) in 2010.

**Supplementary Information:**

The online version contains supplementary material available at 10.1007/s00394-026-04060-1.

## Introduction

Bile acids (BAs) are synthesised in the liver from cholesterol and released into the duodenum via the common bile duct, where they function as surfactant molecules to facilitate lipid digestion. Most BAs are reabsorbed into the hepatic portal vein and enter hepatocytes, completing the enterohepatic circulation [1]. BAs absorbed from the gut, that are not taken up by the liver, are detected in the systemic circulation, while unabsorbed BAs are excreted in faeces [2]. Beyond their role in digestion, BAs serve as signalling molecules that regulate cardiometabolic homeostasis and gastrointestinal function through activation of key receptors such as the Farnesoid X Receptor (FXR) and the G protein-coupled bile acid receptor 1 (TGR5) [1]. Disrupted BA metabolism, characterised by altered BA concentrations in blood and faeces, has been associated with increased risk of various diseases [1].

Dietary fibre has modulatory effects on BA metabolism in humans [3, 4]. Early studies focused primarily on how fibre may protect against colorectal cancer (CRC) by reducing secondary BAs such as deoxycholic acid (DCA) and lithocholic acid (LCA), which are potential risk factors for CRC development [5]. Intervention studies showed that various sources of fibre, such as vegetables [6, 7] and cereal fibre [7–10] increased faecal bulk and lowered secondary BA formation. However, certain soluble and viscous fibres, such as pectin [11] and guar gum [8] have been associated with increased concentrations and excretion of faecal BAs. These findings show that dietary fibres of different physiochemical and fermentation properties may have distinct effects on BA metabolism. The mechanisms underlying these fibre-specific effects may be owing to differential modulation of microbial activity by fibres with varied fermentability [12, 13]. This is particularly relevant to the regulation of BA metabolism, given that BAs undergo multiple microbial transformations in the large intestine, including deconjugation, 7⍺/β-dehydroxylation, oxidation and epimerisation, all of which contribute to BA diversification in the gut [14].

Beyond faecal BAs, circulating BAs have been reported to be associated with the risk of diseases, such as type 2 diabetes and CRC [15–18]. However, current studies investigating the relationship between dietary fibre and circulating BAs are limited and have yielded inconsistent results in healthy people or patients. Byrd et al. reported inverse cross-sectional correlations between dietary fibre intake and circulating concentrations of multiple secondary and glycine and taurine conjugated BAs in a large health cohort [19]. In contrast, another cross-sectional analysis found that concentrations of serum conjugated BAs were higher in vegans compared with omnivores [20]. Heterogeneity also exits in intervention studies. A crossover feeding trial found that a whole-grain diet, compared with a refined-grain diet, increased serum conjugated BAs in healthy adults [21]. However, circulating BA concentrations did not change following high-fibre, high-fruit and vegetable, and low-fat diet interventions [22]. We hypothesise that these discrepancies may be attributed to differences in the types and sources of dietary fibre.

Resistant starch (RS) has been long reported to improve large bowel health, largely due to its high fermentability with beneficial impacts on gut microbiota and metabolites, such as short-chain fatty acids [23]. In contrast, polydextrose (PD) is a type of dietary fibre that is slowly and incompletely fermentable [24]. Both RS and PD are commonly used as food ingredients [23, 24]. Current evidence comparing the effect of highly and slowly fermentable dietary fibres on BA profile remains limited. The current study aimed to use biological samples and gut microbiota data from the Dietary Intervention, Stem cells and Colorectal Cancer (DISC) Study to test the hypothesis that RS and PD have distinct effects on plasma and faecal BA profiles in healthy individuals, which may exhibit fibre-specific associations with gut microbiota.

## Methods

### Intervention study

This study analysed BA profiles in plasma and faecal samples from the DISC Study [25] (ClinicalTrials.gov NCT01214681). Full details of study design have been published [25–27]. In brief, the DISC Study is a double-blind, randomised, placebo-controlled dietary intervention in which 75 healthy participants were supplemented with RS and/or PD, or respective placebo, for 50 days in a 2 × 2 factorial design. Participants were randomised to one of four intervention groups: (A) 23 g Amioca starch (RS placebo) + 12 g maltodextrin (PD placebo); (B) 23 g Amioca starch + 12 g Litesse^®^Ultra™ (90% PD, w/w); (C) 23 g Hi-maize^®^260 (53% type 2 RS, w/w) + 12 g maltodextrin; (D) 23 g Hi-maize^®^260 + 12 g Litesse^®^Ultra™ [25]. The dose of type 2 RS and PD (~ 12 g/day) was determined based on the average daily fibre intake (~ 20 g/day) in the UK [28], with supplementation designed to increase total intake to approximately the recommended 30 g/day. All participants provided informed written consent. Participants were asked to maintain their habitual diet throughout the study. Anthropometric, dietary and lifestyle data and biological samples were collected as described previously [25–27]. Habitual dietary intake was assessed at baseline using a food frequency questionnaire adapted from the EPIC-Norfolk study [26]. Plasma and faecal samples were collected at baseline (day 0) and post-intervention (day 50). Baseline plasma samples from the participants who underwent colonoscopy were fasted as required for endoscopy, whereas those from participants who had flexible sigmoidoscopy were unfasted. There was no difference of the number of fasted baseline samples distributed across four groups. All post-intervention plasma samples were unfasted.

### Gut microbiota 16s rRNA sequencing

Faecal microbiota were analysed using 16 S rRNA gene sequencing as previously described [27]. Briefly, DNA was extracted from 250 mg stool samples using the DNeasy^®^ PowerLyzer^®^ PowerSoil^®^ kit (Qiagen, UK) following the manufacturer’s instructions. The variable region 4 (V4) of the 16 S rRNA gene was amplified and sequenced by NU-OMICS (Northumbria University, UK) according to the Schloss wet-lab MiSeq SOP (Kozich et al., 2013).

### Plasma and faecal bile acid measurement

Plasma BAs were extracted as described previously with adaption [29]. In brief, 80 µl plasma were diluted with 720 µl of ice-cold methanol and incubated in dry ice for 15 min. After centrifugation (16,000rcf, 5 min), the supernatant (600–650 µl) was filtered using a 0.45µM PTFE filter and then evaporated (HT-6, Genevac). The dried extract was reconstituted with 25µL of methanol (≥ 99.8%, ACS reagent, Sigma) with addition of 15 µl of glycochenodeoxycholic acid (GCDCA)-d4 and 15 µl of cholic acid (CA)-d4 at 5ppm each. BAs were extracted from faeces using an adaptation of the method of Zawadzki et al. [30]. Wet faecal samples (20–30 mg) were weighed and added with 720 µl of ice-cold MeOH. The mixture was homogenised using Tissuelyzer (LT, Qiagen). After incubation on dry ice for 1.5 h, the sample was centrifuged, filtered and evaporated. Dried extract was reconstituted with 500µL of methanol. After vortexing, 50µL of 500µL methanol solution was transferred into a glass vial and 30 µl of an internal standard mixture, containing GCDCA-d4, chenodeoxycholic acid (CDCA)-d4, DCA-d4 and LCA-d4 at 5ppm each was added. Faecal samples were weighed, dried in an oven to constant weight, and water content was calculated from weight loss.

BA profiling was performed using ultra-performance liquid chromatography mass spectrometry (Waters Acquity UPLC system and Xevo TQ-S Cronos mass spectrometer, Waters) controlled by MassLynx 4.1 software. Study samples were randomised into experimental batches, with paired baseline and post-intervention samples from each participant analysed within the same batch but in a random order. Quality control (QC) samples, prepared by pooling equal aliquots of study samples, were run with study samples in all batches to monitor inter-batch variation. The limits of quantification (LOQ) were defined by signal-to-noise (SN) ratios of ≥ 10. BAs with more than 30% of samples with concentrations below the LOQ were excluded from quantification. Eleven plasma BAs including CA, CDCA, DCA, GCDCA, glycocholic acid (GCA), taurocholic acid (TCA), taurochenodeoxycholic acid (TCDCA), glycodeoxycholic acid (GDCA), taurodeoxycholic acid (TDCA), glycolithocholic acid (GLCA), glycoursodeoxycholic acid (GUDCA), and ten faecal BAs including CA, CDCA, GCDCA, DCA, LCA, isolithocholic acid (isoLCA), hyodeoxycholic acid (HDCA), 3-ketolithocholic acid (3-keto LCA), 3-ketodeoxycholic acid (3-keto DCA), 12-ketolithocholic acid (12-keto LCA) were quantified. Concentrations below the LOQ were imputed as half the minimum detected value of a given BA [18, 31]. Inter-batch variations (CV) of BAs were between 10.96% and 20.46%, except for plasma glycolithocholic acid (GLCA) (CV = 43.79%) and faecal CDCA (CV = 26.12%). See supplementary methods for full details of calibration sample preparation, QC sample preparation, LC-MS/MS conditions, and analytical performance.

### Statistical analysis

Individual BAs were categorised and summed based on chemical structure (conjugated/unconjugated/keto BAs) or the sequence of BA biotransformation by gut microbiota (primary/secondary BAs), as detailed in Supplementary Table 1. BA concentrations showed right-skewed distributions and were log transformed for analysis of intervention effects and partial correlation.

### Analysis of intervention effects

The general linear model was used to investigate the main intervention effects of RS and PD, as well as the interaction effect (RS * PD) on post-intervention concentrations of individual BAs and BA categories. The model was adjusted for the corresponding baseline BA concentrations, age, BMI, sex, smoking status, habitual intake of alcohol, energy and fibre, endoscopy procedure (that adjusted for baseline fasting status), and experimental LC-MS/MS experiment batch. Least squares means (LSMs) for the main effect were estimated using the *emmeans* function and *p*-values were derived from the contrast of LSMs. For BA proportions and secondary BA transformation ratios DCA/(DCA + CA) and LCA/(LCA+CDCA), which fall within the (0,1) interval, beta regression fitted with a logit link function (*betareg* R package) was used. The same approach was also applied to analyse faecal water content.

### Analysis of partial correlations

Fibre-specific microbial associations with significant BA outcomes at baseline and post-intervention were evaluated among participants who received RS, i.e. RS+ participants, or those who received PD, i.e. PD+ participants. Significant BA outcomes included plasma conjugated BA concentrations to be analysed for RS specific microbial correlations, and faecal primary BA proportions as well as secondary BA transformation ratios for PD specific microbial correlations. A total of 45 taxa at the genus level were included in the analysis, selected based on having a relative abundance greater than 0.01% in at least 50% of samples. To compute pairwise partial correlations, we firstly fitted regression models to adjust covariates for raw relative abundances of taxa and BA outcomes, respectively. Spearman correlations were then performed on the residuals derived from BA models and taxa models. Correlations were adjusted with age, BMI, sex, smoking status, habitual intake of alcohol, energy and fibre, endoscopy procedure, experiment batch and intervention effects (only for post-intervention correlations), i.e., PD adjustment in RS+ participants and vice versa. Details of the statistical approach for partial correlation analysis are provided in Supplementary Table 2. The same approach was also applied to analyse partial correlations between plasma and faecal BA concentrations in response to RS and PD.

The Benjamini–Hochberg method was applied to control the false discovery rate (FDR) for multiple testing on individual BAs in the intervention effect analysis, and on 45 microbial correlations for each BA outcome. Unadjusted p-values were used to evaluate the significance of pre-defined outcomes, including concentrations and proportions of BA categories and secondary BA transformation ratios. Statistical significance was defined as either a raw *p*-value or FDR adjusted p-value (denoted as ‘FDR’) < 0.05, where applicable. All statistical analyses and data visualization were performed using R version 4.4.1.

## Results

### Baseline participant characteristics and BA profile

Plasma BA profiles were obtained from 74 participants with paired samples collected at baseline and post-intervention. The average age and BMI of these participants were 52.5 ± 12.3 years and 30.0 ± 5.3 kg/m². There were no differences in demographic characteristics, macronutrient intake (Supplementary Table 3) or plasma BA concentrations (Supplementary Table 5) between RS − vs. RS + or PD − vs. PD+ participants. RS+ participants had a higher plasma GLCA proportion than RS- participants (*p* = 0.018, and Supplementary Table 6).

Paired faecal samples were available for 50 participants. There were no differences at baseline in demographic characteristics and macronutrient intake between treatment groups (Supplementary Table 4). However, compared with RS- participants at baseline, RS+ participants had lower concentrations of faecal isoLCA, HDCA and 12-keto LCA (*p* < 0.05, Supplementary Table 7) and higher proportions of faecal CA, CDCA and GCDCA (*p* < 0.05, Supplementary Table 8). PD+ participants had higher proportions of faecal LCA and lower proportion of faecal 12-keto LCA compared with PD- participants at baseline (*p* < 0.05, Supplementary Table 8).

### Effects of RS and PD on post-intervention concentrations and proportions of BA in plasma

The post-intervention concentration of total BAs in plasma significantly increased in RS+ participants compared with RS- participants (*p* = 0.003) (Table 1). RS+ participants consistently had higher concentrations of primary BAs (*p* = 0.025), secondary BAs (*p* = 0.0004), glycine conjugated BAs (*p* = 0.002) and taurine conjugated BAs (*p* = 0.003), while the concentrations of unconjugated BAs remained unchanged. For individual BAs, elevated GCA, TCA, GCDCA, TCDCA, GDCA, TDCA, GLCA and GUDCA, as well as DCA concentrations were observed in RS+ compared with RS- participants (all, FDR < 0.05). Regarding BA proportions, increased proportions of glycine conjugated BAs (*p* = 0.032) and decreased proportions of unconjugated BAs (*p* = 0.018) were observed in RS+ participants compared with RS- participants (Supplementary Table 9). In contrast, PD supplementation did not affect concentrations or proportions of plasma BAs (Table 1). No interaction effects between RS and PD on BA concentrations were detected. There was a significant interaction effect on the proportion of taurine conjugated BAs in plasma (p for RS*PD interaction = 0.022, Supplementary Table 9). Additionally, DCA/(DCA + CA) ratio in plasma was unchanged with RS or PD supplementation (Table 3).

**Table 1:** Effects of supplementation with RS and PD on post-intervention concentrations (nmol/L) of BAs in plasma ^a^

BAs/ BA category ^c^	Effect of RS			Effect of PD			*P* value interaction effect
	RS- (*n* = 40)	RS+ (*n* = 34)	*P* value ^b^	PD- (*n* = 37)	PD+ (*n* = 37)	*P* value ^b^	
Total BAs	1847.19 (246.48)	3112.37 (448.85)	0.003	2440.53 (335.73)	2355.69 (333.6)	0.836	0.625
Primary BAs	1230.8 (188.07)	1926.37 (323.43)	0.025	1563.03 (248.66)	1516.91 (247.2)	0.879	0.469
Secondary BAs	556.33 (81.77)	1110.13 (171.48)	0.000	859.73 (127.32)	718.37 (111.78)	0.336	0.710
Glycine conjugated BAs	942 (147.33)	1789.14 (307.8)	0.002	1329.45 (215.75)	1267.73 (212.36)	0.816	0.450
Taurine conjugated BAs	118.29 (16.63)	212.22 (33.12)	0.003	159.46 (23.24)	157.44 (23.87)	0.946	0.172
Unconjugated BAs	670.9 (118.98)	847.34 (151.91)	0.258	812.33 (146.57)	699.81 (124.84)	0.477	0.422
CA	115.9 (33.17)	120.15 (36.17)	0.907	114.93 (33.86)	121.17 (35.82)	0.867	0.646
GCA	137.35 (27.49)	263.15 (55.45)	0.013*	194.99 (40.02)	185.35 (38.61)	0.844	0.550
TCA	24.02 (3.63)	43.62 (6.82)	0.003*	32.03 (4.89)	32.71 (5.13)	0.917	0.116
CDCA	204.33 (48.34)	237.62 (59.89)	0.586	253.07 (62.94)	191.86 (46.58)	0.328	0.657
GCDCA	522.79 (83.05)	902.82 (159.55)	0.011*	700.4 (116.26)	673.87 (114.93)	0.853	0.392
TCDCA	67.84 (9.33)	106.36 (16.71)	0.019*	85.51 (12.26)	84.39 (12.74)	0.943	0.126
DCA	257.78 (39.26)	377.39 (56.99)	0.039*	364.14 (54.88)	267.16 (41.25)	0.097	0.993
GDCA	172.54 (37.3)	419.37 (98.71)	0.002**	279.24 (61.85)	259.12 (60.29)	0.786	0.516
TDCA	22.52 (4.15)	57.14 (11.4)	0.000**	37.97 (7.2)	33.89 (6.64)	0.638	0.270
GLCA	17.33 (2.68)	29.9 (4.22)	0.004*	25.43 (3.77)	20.38 (2.99)	0.228	0.710
GUDCA	67.4 (10.12)	108.62 (17.62)	0.013*	90.04 (13.73)	81.31 (13.17)	0.591	0.845

a. Data are presented as LSMs and (standard error) from analysing using general linear models for the effects of RS or PD supplementation on post-intervention BA concentrations (log transformed). Models were adjusted for the corresponding baseline BA concentrations (log transformed), age, sex, BMI, smoking status, habitual intake of alcohol, energy and fibre, endoscopy procedure and experiment batches

b. P values for the effect of RS and PD were derived from the contrast of LSMs. FDR correction using Benjamini-Hochberg procedure was applied on 11 tests of concentrations of individual plasma BAs. **P value after FDR < 0.01.*P value after FDR < 0.05

c. CA, Cholic acid; GCA, Glycocholic acid; TCA, Taurocholic acid; CDCA, Chenodeoxycholic acid; GCDCA, Glycochenodeoxycholic acid; TCDCA, Taurochenodeoxycholic acid; DCA, Deoxycholic acid; GDCA, Glycodeoxycholic acid; TDCA, Taurodeoxycholic acid; GLCA, Glycolithocholic acid; GUDCA, Glycoursodeoxycholic acid

### Effects of RS and PD on post-intervention concentrations and proportions of BA in faeces

There were no effects of RS and PD supplementation on BA concentrations in wet faeces (all, FDR > 0.05, Supplementary Table 10), although PD nominally decreased the concentration of faecal 12-keto LCA (*p* = 0.023). Faecal water content was not altered by RS and PD (Supplementary Table 11). Regarding BA proportions, RS supplementation significantly decreased the proportion of faecal primary BAs (*p* = 0.032), while PD supplementation significantly enhanced this proportion (*p* = 0.012, Table 2). For individual BAs, RS nominally decreased CA proportion (*p* < 0.05, 0.05 < FDR < 0.1). PD significantly increased CA proportion (FDR = 0.021), and nominally decreased the proportion of isoLCA, 3-keto DCA and 12-keto LCA (all, *p* < 0.05, 0.05 < FDR < 0.1). No interaction effects between RS and PD on BA concentrations or proportions in faeces were detected. Additionally, PD supplementation lowered DCA/(DCA + CA) (*p* = 0.029) and LCA/(LCA+CDCA) (*p* = 0.031) in faeces, whereas RS supplementation had no effect on these two ratios (Table 3).

**Table 2:** Effects of supplementation with RS and PD on post-intervention proportions of BAs (%) in faeces ^a^

BAs/ BA category ^c^	Effect of RS			Effect of PD			*P* value interaction effect
	RS- (*n* = 24)	RS+ (*n* = 26)	*P* value ^b^	PD- (*n* = 22)	PD+ (*n* = 28)	*P* value ^b^	
Primary BAs	2.95 (0.63)	1.66 (0.4)	0.032	1.51 (0.38)	3.1 (0.66)	0.012	0.269
Secondary BAs	79.41 (2.06)	80.78 (1.9)	0.602	81.69 (2.04)	78.49 (2.01)	0.244	0.169
Keto BAs	15.87 (1.35)	17.15 (1.21)	0.454	17.32 (1.4)	15.71 (1.18)	0.352	0.434
CA	0.85 (0.18)	0.41 (0.1)	0.008	0.34 (0.08)	0.92 (0.2)	0.002*	0.989
CDCA	1.39 (0.32)	0.99 (0.25)	0.207	0.91 (0.25)	1.47 (0.32)	0.084	0.061
GCDCA	0.19 (0.03)	0.17 (0.02)	0.547	0.15 (0.03)	0.2 (0.02)	0.166	0.138
DCA	29.42 (2.47)	26.75 (2.12)	0.387	29.06 (2.45)	27.11 (2.06)	0.51	0.275
LCA	37.77 (2.38)	41.72 (2.29)	0.215	40.36 (2.4)	39.12 (2.22)	0.69	0.467
IsoLCA	6.22 (0.69)	7.62 (0.71)	0.155	8.1 (0.78)	5.73 (0.56)	0.011	0.482
HDCA	5.1 (0.48)	4.87 (0.41)	0.705	5.36 (0.49)	4.61 (0.36)	0.194	0.534
3-keto DCA	0.89 (0.09)	0.85 (0.07)	0.723	0.98 (0.09)	0.76 (0.06)	0.025	0.051
3-keto LCA	4.13 (0.62)	3.81 (0.54)	0.662	3.85 (0.59)	4.09 (0.58)	0.754	0.786
12-keto LCA	10.69 (0.87)	12.14 (0.77)	0.186	12.55 (0.92)	10.28 (0.71)	0.04	0.329

a. Data are present as estimated marginal means and (standard error) from analysing using beta regression models for the effects of RS or PD supplementation on post-intervention BA proportions. Models were adjusted for the corresponding baseline BA proportions, age, sex, BMI, smoking status, habitual intake of alcohol, energy and fibre, endoscopy procedure and experiment batches

b.* P* values for the effect of RS and PD were derived from the contrast of estimated marginal means. FDR correction using Benjamini-Hochberg procedure was applied on 10 tests of proportions of individual faecal BAs. *P value after FDR < 0.05

c. CA, Cholic acid; CDCA, Chenodeoxycholic acid; GCDCA, Glycochenodeoxycholic acid; DCA, Deoxycholic acid; isoLCA, Isolithocholic acid; HDCA, Hyodeoxycholic acid; LCA, Lithocholic acid; 3-keto LCA, 3-ketolithocholic acid; 3-keto DCA, 3-ketodeoxycholic acid;12-keto LCA, 12-ketolithocholic acid

**Table 3:** Effects of supplementation with RS and PD on post-intervention secondary BA transformation ratios in plasma and faeces ^a^

Secondary BA transformation ratios ^c^	Effect of RS			Effect of PD			*P* value interaction effect
	RS-	RS+	*P* value ^b^	PD-	PD+	*P* value ^b^	
Plasma DCA / (DCA + CA)	0.66 (0.03)	0.73 (0.03)	0.127	0.73 (0.03)	0.67 (0.03)	0.170	0.338
Faecal DCA / (DCA + CA)	0.96 (0.01)	0.97 (0.01)	0.161	0.97 (0.01)	0.95 (0.01)	0.029*	0.269
Faecal LCA / (LCA+CDCA)	0.96 (0.01)	0.97 (0.01)	0.069	0.98 (0.01)	0.96 (0.01)	0.031*	0.126

a. Data are present as estimated marginal means and (standard error) from analysing using beta regression models for the effects of RS or PD supplementation on post-intervention secondary BA transformation ratios. Models were adjusted for the corresponding baseline ratios, age, sex, BMI, smoking status, habitual intake of alcohol, energy and fibre, endoscopy procedure and experiment batches

b.* P* values for the effect of RS and PD were derived from the contrast of estimated marginal means. *P value < 0.05

c. CA, Cholic acid; CDCA, Chenodeoxycholic acid; DCA, Deoxycholic acid; LCA, Lithocholic acid

### Associations between plasma BAs and faecal BAs in response to RS and PD supplementation

**Fig. 1:**
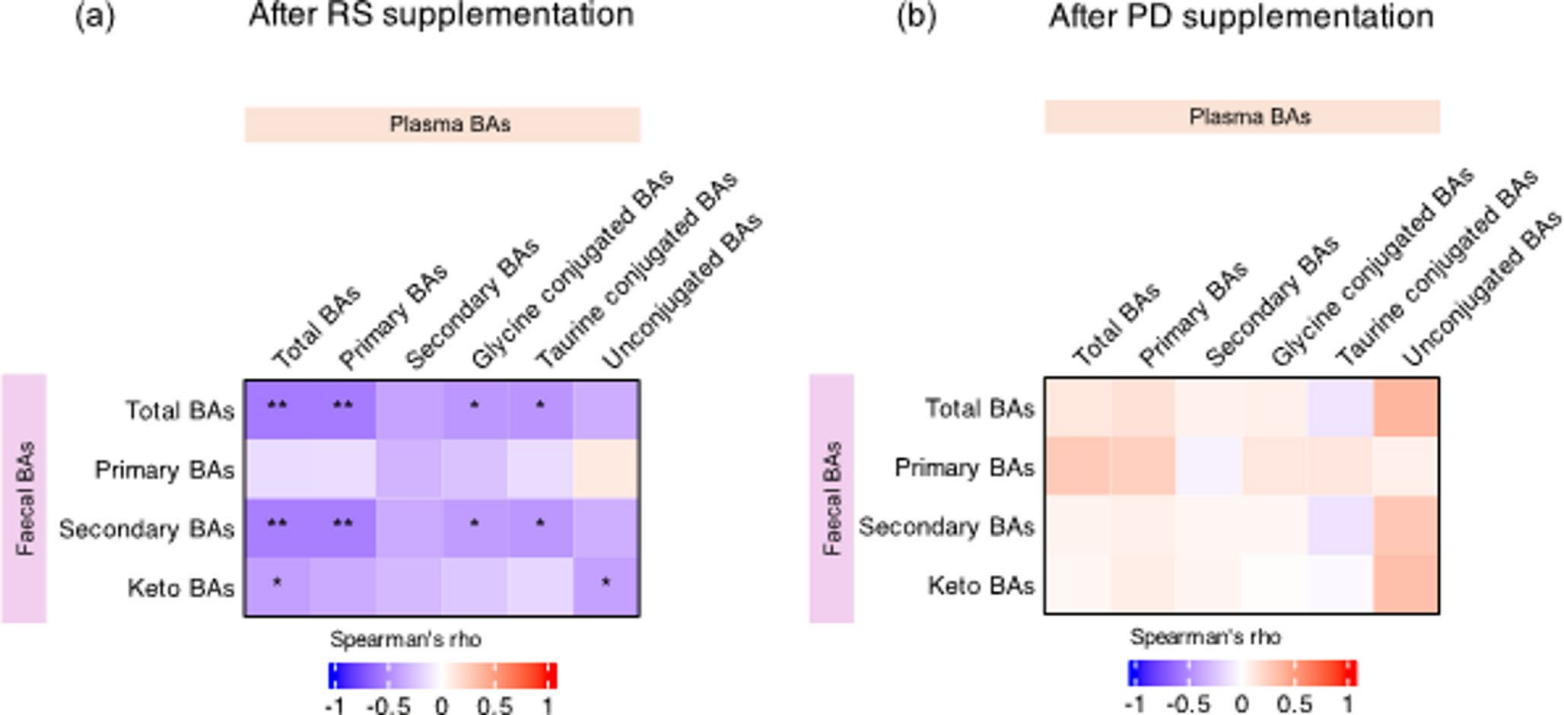
Partial spearman correlations between concentrations of BAs in plasma and faeces** a** after RS supplementation (RS+ participants, *n* = 25) and** b** after PD supplementation (PD+ participants, *n* = 27). Spearman correlations were adjusted for treatment (PD adjustment in RS+ participants and vice versa), age, BMI, sex, smoking status, habitual intake of alcohol, energy and fibre, experimental batch. *Unadjusted* p* value < 0.05, **Unadjusted* p* value < 0.01

We hypothesised that the distinct effect of RS and PD on plasma and faecal BAs may lead to differential inter-matrix correlations between plasma BAs and faecal BAs. Partial correlation analysis showed widespread negative correlations between faecal BAs and plasma BAs after RS supplementation (Fig. 1a), particularly for faecal secondary BAs with plasma primary BAs and conjugated BAs (*p* < 0.01). These negative correlations were not observed at baseline (Supplementary Fig. 1a). In contrast, the concentrations of plasma BAs and faecal BAs were not associated after PD supplementation (Fig. 1b).

### Associations between gut microbiota and plasma conjugated BAs in response to RS supplementation

**Fig. 2:**
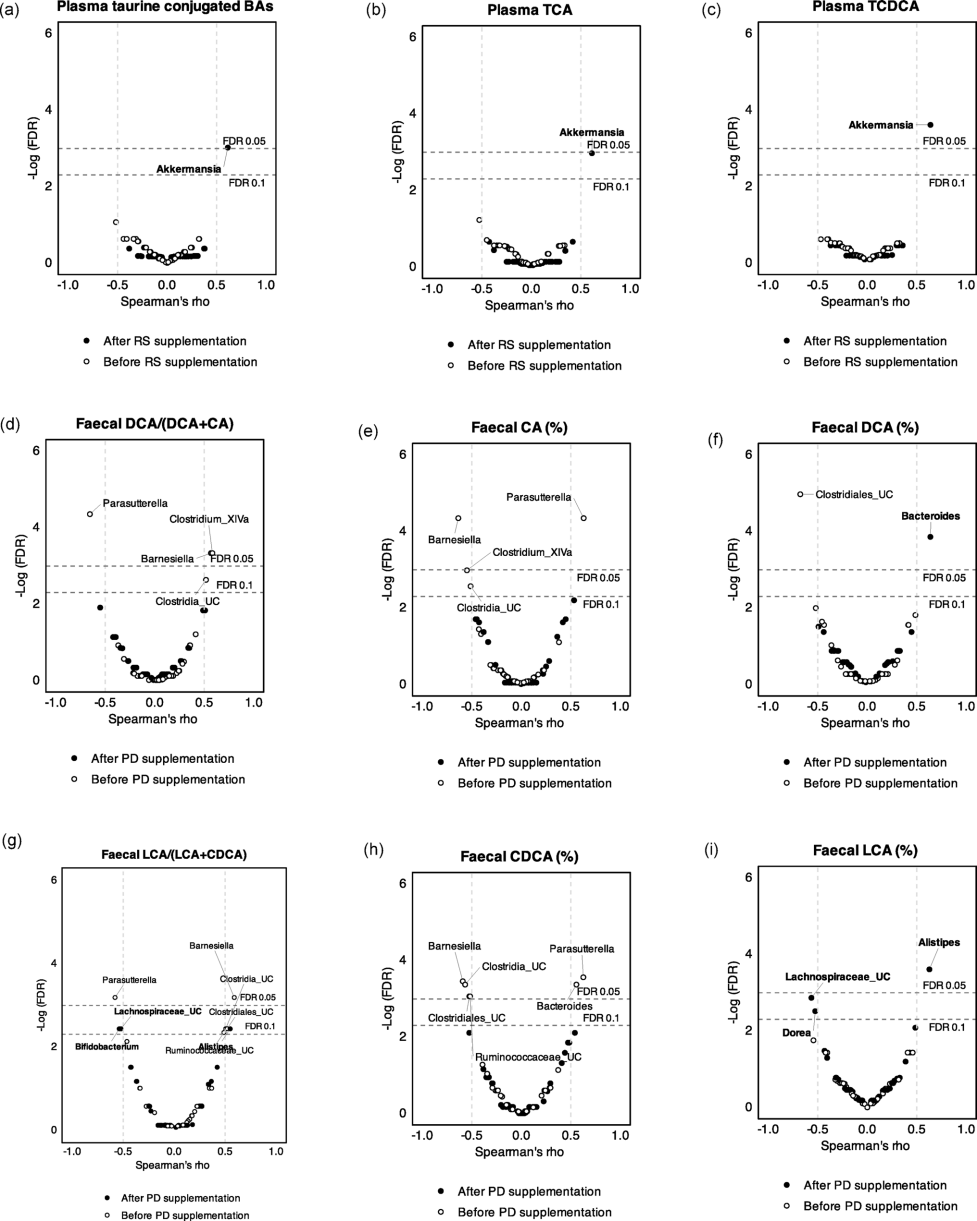
Partial spearman correlations between the relative abundance of genus abundance and (**a**–**c**) concentrations of plasma BAs in response to RS (RS+ participants, *n* = 26) and (**d**–**i**) proportions or ratios of faecal BAs in response to PD (PD+ participants, *n* = 26). FDR correction was applied on 45 genera for each BA outcome.* Abbreviations*: TCA, taurocholic acid; TCDCA, taurochenodeoxycholic acid; CA, cholic acid; CDCA, chenodeoxycholic acid; DCA, deoxycholic acid; LCA, lithocholic acid

A wide range of bacteria in the human gut express bile salt hydrolase (BSH), which deconjugates glycine or taurine conjugated BAs to liberate unconjugated BAs [14]. Consequently, we hypothesised that RS supplementation would alter the microbial associations of plasma conjugated BAs, specifically showing negative correlations between these BAs and BA-deconjugating bacteria such as *Bacteroides*, *Lactobacillus*, and *Bifidobacterium* after RS supplementation [14]. However, partial correlation analysis showed no negative correlations between these three genera and conjugated BAs at baseline or post intervention (Supplementary Table 12). Notably, in RS+ participants post intervention, the abundance of *Akkermansia* showed strong positive correlations with taurine primary conjugated BAs (rho = 0.61, FDR = 0.049), including TCDCA (rho = 0.64, FDR = 0.027) and TCA (rho = 0.61, FDR = 0.051) (Fig. 2a and c). Additionally, *Akkermansia* was nominally positively correlated with TDCA and several glycine conjugated BAs (*p* < 0.05, Supplementary Table 13). These post-intervention positive associations between *Akkermansia* and plasma conjugated BAs were not observed at baseline (Supplementary Table 13).

### Associations between gut microbiota and faecal secondary BA transformation in response to PD supplementation

The microbial transformation of primary BAs to secondary BAs via 7α-dehydroxylation is primarily mediated by *Clostridium* species [32]. In participants following PD supplementation, we hypothesised that the microbial associations of faecal secondary BA transformation ratios would be altered, particularly with *Clostridium* species. Partial correlation analysis revealed that PD supplementation substantially changed the microbial correlations with DCA/(DCA + CA) and LCA/(LCA+CDCA). At baseline, DCA/(DCA + CA) correlated positively with *Clostridium XIVa* and *Barnesiella*, and negatively with *Parasutterella* (FDR < 0.05, Fig. 2d), mirroring their correlation with CA proportion (Fig. 2e). However, these associations were no longer present after PD supplementation. Similarly, LCA/(LCA+CDCA) was positively correlated with *Barnesiella* and negatively correlated with *Parasutterella* at baseline (FDR < 0.05, Fig. 2g), but these associations were no longer present after PD supplementation. Instead, post-intervention LCA/(LCA+CDCA) tended to be negatively associated with *Bifidobacterium* (rho= − 0.54, FDR = 0.088) and *Lachnospiraceae_UC* (rho= − 0.52, FDR = 0.088), and positively associated with *Alistipes* (rho = 0.55, FDR = 0.088). These associations partially reflected their relationships with LCA (Fig. 2i).

## Discussion

We observed that supplementation with RS, but not PD, significantly increased circulating BA concentrations, with increases in conjugated BAs including GCA, GCDCA, GDCA, GLCA, GUDCA, TCA, TCDCA, TDCA, and DCA. Concentrations of taurine conjugated BAs were correlated positively with *Akkermansia* abundance after RS supplementation. Neither RS nor PD altered BA concentrations in wet faeces, but RS decreased and PD increased the proportion of faecal primary BAs. Moreover, PD specifically reduced secondary BA transformation ratios DCA/(DCA + CA) and LCA/(LCA+CDCA). The specific microbiota correlated with these ratios shifted following PD supplementation.

To date, few studies have reported the effects of RS and PD on circulating BAs and the findings were inconsistent. Bush et al. found that supplementation with resistant potato starch containing 2.1 g/day RS2 for 4 weeks in healthy participants lowered taurine conjugated BAs in serum with no changes in total BAs or in glycine conjugated BAs [33]. We speculate that the findings from Bush et al. differed from those in our study because of a lower RS2 dose and a shorter intervention duration. In contrast, Li et al. reported that supplementation with 40 g RS2/day for 8 weeks increased the concentrations of serum GDCA, GCA, DCA, 7-ketolithocholic acid and TDCA in overweight/obese participants [34]. Similarly, Dhakal et al. reported increased circulating conjugated BAs and DCA in participants with metabolic syndrome supplemented with 12 g/day RS4 for 12 weeks [35]. The intervention intensity of these studies, including ours, was ranked as Bush et al. (2.1 g/day RS2 for 4 weeks) < the DISC Study (12 g/day RS2 for 7 weeks) < Dhakal et al. (12 g/day RS4 for 12 weeks) and Li et al. (40 g/day RS2 for 8 weeks). This suggests that higher RS exposure is more likely to increase circulating BAs, potentially independent of metabolic status. Further human studies are warranted to investigate circulating BA responses to different dose of RS supplementation across metabolically healthy and unhealthy populations. Regarding PD, we are aware of only one human study that reported no effect of supplementation with 12 g/day PD in healthy participants with obesity for 6 months on plasma BAs [36]. This trial used the same type and dose of PD as in our study, supporting our finding of no effects of PD on circulating BAs.

Most conjugated BAs are actively reabsorbed in the terminal ileum, while a smaller portion escapes reabsorption and reaches the colon, where gut microbiota expressing BSH catalyse deconjugation [14]. Therefore, we initially hypothesised that RS increases circulating conjugated BAs by reducing microbial deconjugation. However, the current study did not identify negative correlations between plasma conjugated BAs and the commonly reported BA-deconjugating bacteria including *Bacteroides*, *Lactobacillus*, and *Bifidobacterium* in response to RS. This suggests that increased plasma conjugated BA in response to RS was not directly related to the abundance of individual BA-deconjugating bacteria in faeces. One possible explanation is that ~ 95% of conjugated BAs are reabsorbed in the terminal ileum before reaching the large intestine [37], thereby possibly weakening the associations with faecal gut microbiota. Thus, it may be important to investigate the impact of the ileal microbiota on circulating conjugated BAs. Nevertheless, microbial BSH activity and BA deconjugation profile were reported to be similar between the proximal colon and faeces [38, 39], suggesting that faecal microbiota may still provide relevant insights. Another potential reason is that deconjugation likely reflects activity across a broader microbial community rather than specific taxa, as a large group of bacterial species have BSH-expressing ability [40], Supporting this concept, Li et al. reported that RS supplementation reduced faecal metagenomic expression of BSH, accompanied by increased circulating conjugated BAs [34]. In addition to the commonly reported BA-deconjugating bacteria, we observed positive correlations between taurine conjugated BAs and *Akkermansia* in response to RS. *Akkermansia* is not typically considered a BA-metabolising bacteria, but animal studies have reported that *Akkermansia* influenced the BA pool and increased taurine conjugated BAs in plasma [41, 42]. Taurine conjugated BAs have specifically greater agonist potency for the TGR5 receptor [43]. Whether the observed associations between taurine conjugated BAs and *Akkermansia* are involved with alternations in TGR5 activation with RS supplementation warrants further investigation.

In addition to the microbial factor, another hypothesis for increased circulating BAs induced by RS is that RS may enhance intestinal reabsorption of conjugated BAs into the portal vein and/or reducing hepatic BA uptake. Supporting this concept, our correlation analysis between plasma BAs and faecal BAs suggests that increases in plasma BAs were associated with reallocation of BAs from faeces in response to RS. Animal evidence has shown that RS stimulated ileal absorption of conjugated BAs [44] and downregulated gene expression of hepatic BA uptake transporters [45]. However, it remains unclear whether RS directly, or RS-derived metabolites, e.g., SCFAs, regulate transporter-mediated flux of conjugated BAs. Furthermore, viscosity of fibres is considered as an important factor influencing intestinal BA reabsorption [4]. Higher viscosity contributes to BA-binding of dietary fibre in the intestine, which is associated with interrupted intestinal BA absorption and increased faecal BA excretion [4]. Unfortunately, because we do not have data on faecal daily output, we do not know whether RS and PD affected faecal BA excretion. However, both RS and PD are both non-viscous fibres, therefore, their BA-binding ability in the intestine and contribution to faecal BA excretion is not expected to differ substantially. Additionally, some animal evidence showed that RS influenced hepatic BA synthesis [45] and conjugation [46]. BA biosynthesis is negatively regulated by BAs across gut-liver axis via FXR signalling [1]. Whether RS-induced increases in circulating BAs reflect an enlarged BA pool or contribute to negative feedback regulation on BA synthesis warrants further investigation.

Although RS supplementation markedly increased BA concentrations in plasma, neither RS nor PD altered total or individual BA concentrations in wet faeces. Faecal water content was not altered by RS and PD, therefore, faecal BA concentrations measured in wet faeces were not expected to be affected by this variation. This aligns with the finding from Dhakal et al., who also observed increased plasma BAs with RS4 supplementation without changes in BA concentrations analysed in wet faeces [35]. In contrast, other studies have reported that the supplementation of RS [47–49] and PD [50] reduced secondary BAs in dry faeces and in faecal water. When assessing molar proportions of BAs, PD increased faecal proportion of primary BAs and decreased the ratios of DCA/(DCA + CA) and LCA/(LCA + CDCA). In addition, baseline positive associations between 7⍺-dehydroxylating bacteria, such as *Clostridium* clusters *XIVa* and secondary BA transformation ratios were shifted after PD supplementation. This suggests that the reduction of PD on secondary BA transformation was associated with BA-dehydroxylating bacteria. This notion is supported by decreased faecal proportions of isoLCA, 3-keto DCA, and 12-keto LCA, which are derived from LCA and DCA through microbial oxidation and epimerization, largely mediated by *Clostridium* species [51]. On contrary, RS decreased the proportion of primary BAs in faeces, and its effect on increasing the LCA/(LCA+CDCA) ratio approached significance. A plausible explanation for the divergent effects of RS and PD lies in their fermentation kinetics: RS is rapidly fermented in the proximal colon, while PD is more slowly fermented, leading to SCFA accumulation primarily in the distal colon. As BA-dehydroxylating bacteria prefer an alkaline luminal environment approaching the distal colon [52], the acidification caused by distal SCFAs accumulation from PD may suppress their growth. Supporting this, we observed negative correlations between LCA/(LCA + CDCA) and SCFA-producing genera, *Bifidobacterium*, *Lachnospiraceae_UC*. These genera are not directly involved in 7⍺-dehydroxylation but may indirectly inhibit this process through luminal acidification [53].

Further research is needed to investigate the health implication of RS-induced increases in circulating conjugated BAs and DCA. A human trial has shown that ileo-colonic delivery of conjugated BAs improved glucose homeostasis via colonic GLP-1-producing enteroendocrine cells, potentially mediated by TGR5 activation [54]. Whether increased circulating conjugated BAs by RS confers similar benefits warrants further investigation, particularly in pre-diabetic individuals. In addition, prospective studies reported that circulating DCA and conjugated BAs were associated with increased CRC risk [15, 18]. Although DCA is considered a carcinogenic metabolite in the context of a high-fat diet [55], it remains unclear whether long-term RS-induced increases in these circulating BAs elevate CRC risk in healthy individuals or individuals with high CRC risk.

A strength of this study is that we compared two different types of dietary fibre at matched doses and durations, minimising confounding from dosage and intervention duration. Additionally, simultaneous quantification of BAs in plasma and faeces allows an integrated view of BA dynamics across intestinal and systemic compartments. The use of unfasted post-intervention plasma samples reflects real-life metabolic responses. To account for individual variation of habitual diet, we included habitual intake of alcohol, energy and fibre as covariates in the statistical models. A limitation is that this study is a secondary analysis without a priori sample size calculation, which may limit statistical power. A further limitation is that GCDCA was the only conjugated BA in faeces that was above LOQ, restricting our ability to explore links between conjugated BAs in plasma and faeces.

## Conclusion

In conclusion, RS and PD supplementation in healthy individuals had distinct effects on BA profiles in plasma and faeces. RS supplementation increased plasma BAs, particularly conjugated BAs. Taurine primary conjugated BAs showed positive correlations with *Akkermansia* in response to RS. In contrast, PD supplementation decreased secondary BA transformation ratios DCA/(DCA + CA) and LCA/(LCA+CDCA) in faeces, which may be associated with inhibited microbial 7α-dehydroxylation activity. These fibre-specific effects on BA profile and microbial associations likely reflect the different fermentation properties of RS and PD. Further studies are needed to evaluate the health consequences of RS-induced increases in circulating BAs, particularly in relation to glucose metabolism and CRC risk. Investigation of BA metabolism, particularly circulating BA profiles, warrants extension to other dietary fibres with varying viscosity and fermentability.

## Supplementary Information

Below is the link to the electronic supplementary material.

Supplementary Material 1

## Data Availability

The data supporting the conclusion of this article is published on Figshare 10.6084/m9.figshare.32117815.

## References

[1] Fleishman JS, Kumar S (2024) Bile acid metabolism and signaling in health and disease: molecular mechanisms and therapeutic targets. Sig Transduct Target Ther 9:1–51. 10.1038/s41392-024-01811-6 10.1038/s41392-024-01811-6 PMC11045871 38664391

[2] Stellaard F, Lütjohann D (2021) Dynamics of the enterohepatic circulation of bile acids in healthy humans. Am J Physiol-Gastrointest Liver Physiol 321:G55–G66. 10.1152/ajpgi.00476.2020 33978477 10.1152/ajpgi.00476.2020

[3] Singh J, Metrani R, Shivanagoudra SR et al (2019) Review on bile acids: effects of the gut microbiome, interactions with dietary fiber, and alterations in the bioaccessibility of bioactive compounds. J Agric Food Chem 67:9124–9138. 10.1021/acs.jafc.8b07306 30969768 10.1021/acs.jafc.8b07306

[4] Naumann S, Haller D, Eisner P, Schweiggert-Weisz U (2020) Mechanisms of interactions between bile acids and plant compounds—a review. Int J Mol Sci 21:6495. 10.3390/ijms21186495 32899482 10.3390/ijms21186495 PMC7555273

[5] Farhana L, Nangia-Makker P, Arbit E et al (2016) Bile acid: a potential inducer of colon cancer stem cells. Stem Cell Res Ther 7:181. 10.1186/s13287-016-0439-4 27908290 10.1186/s13287-016-0439-4 PMC5134122

[6] Fechner A, Fenske K, Jahreis G (2013) Effects of legume kernel fibres and citrus fibre on putative risk factors for colorectal cancer: a randomised, double-blind, crossover human intervention trial. Nutr J 12:101. 10.1186/1475-2891-12-101 24060277 10.1186/1475-2891-12-101 PMC3717035

[7] Lampe JW, Slavin JL, Melcher EA, Potter JD (1992) Effects of cereal and vegetable fiber feeding on potential risk factors for colon cancer. Cancer Epidemiol Biomarkers Prev 1:207–211 1339081

[8] Thompson MH, Owen RW, Hill MJ, Cummings JH (1985) Factors affecting faecal bile acid concentrations: effect of fat and fibre. Biochem Soc Trans 13:392–392. 10.1042/bst0130392

[9] Walters RL, Baird IM, Davies PS et al (1975) Effects of two types of dietary fibre on faecal steroid and lipid excretion. Br Med J 2:536–538. 10.1136/bmj.2.5970.536 1097036 10.1136/bmj.2.5970.536 PMC1673356

[10] Hakkola S, Nylund L, Rosa-Sibakov N et al (2021) Effect of oat β-glucan of different molecular weights on fecal bile acids, urine metabolites and pressure in the digestive tract—a human cross over trial. Food Chem 342:128219. 10.1016/j.foodchem.2020.128219 33077284 10.1016/j.foodchem.2020.128219

[11] Hillman LC, Peters SG, Fisher CA, Pomare EW (1986) Effects of the fibre components pectin, cellulose, and lignin on bile salt metabolism and biliary lipid composition in man. Gut 27:29–36. 10.1136/gut.27.1.29 3005138 10.1136/gut.27.1.29 PMC1433181

[12] Baxter NT, Schmidt AW, Venkataraman A et al (2019) Dynamics of human gut microbiota and short-chain fatty acids in response to dietary interventions with three fermentable fibers. mBio 10:e02566–e02518. 10.1128/mBio.02566-18 30696735 10.1128/mBio.02566-18 PMC6355990

[13] Cummings JH, Macfarlane GT, Englyst HN (2001) Prebiotic digestion and fermentation123. Am J Clin Nutr 73:415s–420s. 10.1093/ajcn/73.2.415s 11157351 10.1093/ajcn/73.2.415s

[14] Ridlon JM, Kang D-J, Hylemon PB (2006) Bile salt biotransformations by human intestinal bacteria. J Lipid Res 47:241–259. 10.1194/jlr.R500013-JLR200 16299351 10.1194/jlr.R500013-JLR200

[15] Kühn T, Stepien M, López-Nogueroles M et al (2020) Prediagnostic plasma bile acid levels and colon cancer risk: a prospective study. J Natl Cancer Inst 112:516–524. 10.1093/jnci/djz166 31435679 10.1093/jnci/djz166 PMC7225675

[16] Ahmad TR, Haeusler RA (2019) Bile acids in glucose metabolism and insulin signalling—mechanisms and research needs. Nat Rev Endocrinol 15:701–712. 10.1038/s41574-019-0266-7 31616073 10.1038/s41574-019-0266-7 PMC6918475

[17] Qi L, Chen Y (2023) Circulating bile acids as biomarkers for disease diagnosis and prevention. J Clin Endocrinol Metab 108:251–270. 10.1210/clinem/dgac659 36374935 10.1210/clinem/dgac659

[18] Loftfield E, Falk RT, Sampson JN et al (2022) Prospective associations of circulating bile acids and short-chain fatty acids with incident colorectal cancer. JNCI Cancer Spectr 6:pkac027. 10.1093/jncics/pkac027 35583137 10.1093/jncics/pkac027 PMC9115675

[19] Byrd DA, Sinha R, Weinstein SJ et al (2021) An investigation of cross-sectional associations of a priori–selected dietary components with circulating bile acids. Am J Clin Nutr 114:1802–1813. 10.1093/ajcn/nqab232 34477829 10.1093/ajcn/nqab232 PMC8574696

[20] Trefflich I, Marschall H-U, di Giuseppe R et al (2020) Associations between dietary patterns and bile acids—Results from a cross-sectional study in vegans and omnivores. Nutrients 12:47. 10.3390/nu12010047 10.3390/nu12010047 PMC7019893 31878000

[21] Ginos BNR, Navarro SL, Schwarz Y et al (2018) Circulating bile acids in healthy adults respond differently to a dietary pattern characterized by whole grains, legumes and fruits and vegetables compared to a diet high in refined grains and added sugars: a randomized, controlled, crossover feeding study. Metabolism 83:197–204. 10.1016/j.metabol.2018.02.006 29458053 10.1016/j.metabol.2018.02.006 PMC5960615

[22] Byrd DA, Gomez M, Hogue S et al (2022) Circulating bile acids and adenoma recurrence in the context of adherence to a high-fiber, high-fruit and vegetable, and low-fat dietary intervention. Clin Transl Gastroenterol 13:e00533. 10.14309/ctg.0000000000000533 36113023 10.14309/ctg.0000000000000533 PMC9624497

[23] DeMartino P, Cockburn DW (2020) Resistant starch: impact on the gut microbiome and health. Curr Opin Biotechnol 61:66–71. 10.1016/j.copbio.2019.10.008 31765963 10.1016/j.copbio.2019.10.008

[24] Röytiö H, Ouwehand AC (2014) The fermentation of polydextrose in the large intestine and its beneficial effects. Benef Microbes 5:305–313. 10.3920/BM2013.0065 24736314 10.3920/BM2013.0065

[25] Malcomson FC, Willis ND, McCallum I et al (2017) Effects of supplementation with nondigestible carbohydrates on fecal calprotectin and on epigenetic regulation of *SFRP1* expression in the large-bowel mucosa of healthy individuals1,2. Am J Clin Nutr 105:400–410. 10.3945/ajcn.116.135657 28077379 10.3945/ajcn.116.135657 PMC5267298

[26] Malcomson FC, Willis ND, McCallum I et al (2020) Resistant starch supplementation increases crypt cell proliferative state in the rectal mucosa of older healthy participants. Br J Nutr 124:374–385. 10.1017/S0007114520001312 32279690 10.1017/S0007114520001312 PMC7369377

[27] Malcomson FC, Louca P, Nelson A et al (2024) Effects of non-digestible carbohydrates on gut microbiota and microbial metabolites: a randomised, controlled dietary intervention in healthy individuals. Br J Nutr 132:1433–1445. 10.1017/S000711452400271X 39494600 10.1017/S000711452400271X PMC7616798

[28] Gressier M, Frost G (2022) Minor changes in fibre intake in the UK population between 2008/2009 and 2016/2017. Eur J Clin Nutr 76:322–327. 10.1038/s41430-021-00933-2 33986495 10.1038/s41430-021-00933-2 PMC8821000

[29] Connell E, Sami S, Khondoker M et al (2024) Circulatory dietary and gut-derived metabolites predict preclinical Alzheimer’s disease. medRxiv. 10.1101/2024.05.10.24307050.

[30] Zawadzki AD, Thiele M, Suvitaival T et al (2022) High-throughput UHPLC-MS to screen metabolites in feces for gut metabolic health. Metabolites 12:211. 10.3390/metabo12030211 35323654 10.3390/metabo12030211 PMC8950041

[31] Farhat Z, Sampson JN, Hildesheim A et al (2021) Reproducibility, temporal variability, and concordance of serum and fecal bile acids and short chain fatty acids in a population-based study. Cancer Epidemiol Biomarkers Prev 30:1875–1883. 10.1158/1055-9965.EPI-21-0361 34376486 10.1158/1055-9965.EPI-21-0361 PMC8608567

[32] Cai J, Rimal B, Jiang C et al (2022) Bile acid metabolism and signaling, the microbiota, and metabolic disease. Pharmacol Ther 237:108238. 10.1016/j.pharmthera.2022.108238 35792223 10.1016/j.pharmthera.2022.108238

[33] Bush JR, Iwuamadi I, Han J et al (2024) Resistant potato starch supplementation reduces serum free fatty acid levels and influences bile acid metabolism. Metabolites 14:536. 10.3390/metabo14100536 39452917 10.3390/metabo14100536 PMC11510092

[34] Li H, Zhang L, Li J et al (2024) Resistant starch intake facilitates weight loss in humans by reshaping the gut microbiota. Nat Metab 6:578–597. 10.1038/s42255-024-00988-y 38409604 10.1038/s42255-024-00988-y PMC10963277

[35] Dhakal S, Dey M (2022) Resistant starch type-4 intake alters circulating bile acids in human subjects. Front Nutr 9:930414. 10.3389/fnut.2022.930414 36337613 10.3389/fnut.2022.930414 PMC9631925

[36] Hibberd AA, Yde CC, Ziegler ML et al (2019) Probiotic or synbiotic alters the gut microbiota and metabolism in a randomised controlled trial of weight management in overweight adults. Benef Microbes 10:121–136. 10.3920/BM2018.0028 30525950 10.3920/BM2018.0028

[37] Murakami M, Iwamoto J, Honda A et al (2018) Detection of gut dysbiosis due to reduced clostridium subcluster XIVa using the fecal or serum bile acid profile. Inflamm Bowel Dis 24:1035–1044. 10.1093/ibd/izy022 29688473 10.1093/ibd/izy022

[38] Thomas LA, Veysey MJ, French G et al (2001) Bile acid metabolism by fresh human colonic contents: a comparison of caecal versus faecal samples. Gut 49:835–842. 10.1136/gut.49.6.835 11709519 10.1136/gut.49.6.835 PMC1728526

[39] Chinda D, Takada T, Mikami T et al (2022) Spatial distribution of live gut microbiota and bile acid metabolism in various parts of human large intestine. Sci Rep 12:3593. 10.1038/s41598-022-07594-6 35246580 10.1038/s41598-022-07594-6 PMC8897406

[40] Jones BV, Begley M, Hill C et al (2008) Functional and comparative metagenomic analysis of bile salt hydrolase activity in the human gut microbiome. Proc Natl Acad Sci 105:13580–13585. 10.1073/pnas.0804437105 18757757 10.1073/pnas.0804437105 PMC2533232

[41] Wu W, Kaicen W, Bian X et al (2023) Akkermansia muciniphila alleviates high-fat-diet-related metabolic-associated fatty liver disease by modulating gut microbiota and bile acids. Microb Biotechnol 16:1924–1939. 10.1111/1751-7915.14293 37377410 10.1111/1751-7915.14293 PMC10527187

[42] Grajeda-Iglesias C, Durand S, Daillère R et al (2021) Oral administration of Akkermansia muciniphila elevates systemic antiaging and anticancer metabolites. Aging 13:6375–6405. 10.18632/aging.202739 33653967 10.18632/aging.202739 PMC7993698

[43] Sato H, Macchiarulo A, Thomas C et al (2008) Novel potent and selective bile acid derivatives as TGR5 agonists: biological screening, structure–activity relationships, and molecular modeling studies. J Med Chem 51:1831–1841. 10.1021/jm7015864 18307294 10.1021/jm7015864

[44] Riottot M, Sacquet E (1985) Increase in the ileal absorption rate of sodium taurocholate in germ-free or conventional rats given an amylomaize-starch diet. Br J Nutr 53:307–310. 10.1079/BJN19850038 4063275 10.1079/bjn19850038

[45] Wang Z, Zhan C, Zhang Y et al (2023) Dietary resistant starch regulates bile acid metabolism by modulating the FXR/LRH-1 signaling pathway in broilers. Agriculture 13:2159. 10.3390/agriculture13112159

[46] Trautwein EA, Forgbert K, Rieckhoff D, Erbersdobler HF (1999) Impact of β-cyclodextrin and resistant starch on bile acid metabolism and fecal steroid excretion in regard to their hypolipidemic action in hamsters. Biochim Biophys Acta BBA - Mol Cell Biol Lipids 1437:1–12. 10.1016/S0005-2760(98)00174-X 10.1016/s0005-2760(98)00174-x 9931405

[47] Hylla S, Gostner A, Dusel G et al (1998) Effects of resistant starch on the colon in healthy volunteers: possible implications for cancer prevention. Am J Clin Nutr 67:136–142. 10.1093/ajcn/67.1.136 9440388 10.1093/ajcn/67.1.136

[48] van Munster IP, Tangerman A, Nagengast FM (1994) Effect of resistant starch on colonic fermentation, bile acid metabolism, and mucosal proliferation. Dig Dis Sci 39:834–842. 10.1007/BF02087431 8149850 10.1007/BF02087431

[49] Grubben MJ, van den Braak CC, Essenberg M et al (2001) Effect of resistant starch on potential biomarkers for colonic cancer risk in patients with colonic adenomas. Dig Dis Sci 46:750–756. 10.1023/A:1010787931002 11330408 10.1023/a:1010787931002

[50] Hengst C, Ptok S, Roessler A et al (2009) Effects of polydextrose supplementation on different faecal parameters in healthy volunteers. Int J Food Sci Nutr 60:96–105. 10.1080/09637480802526760 19107626 10.1080/09637480802526760

[51] Fiorucci S, Carino A, Baldoni M et al (2021) Bile acid signaling in inflammatory bowel diseases. Dig Dis Sci 66:674–693. 10.1007/s10620-020-06715-3 33289902 10.1007/s10620-020-06715-3 PMC7935738

[52] Brinck JE, Sinha AK, Laursen MF et al (2025) Intestinal pH: a major driver of human gut microbiota composition and metabolism. Nat Rev Gastroenterol Hepatol 22:639–656. 10.1038/s41575-025-01092-6 40603778 10.1038/s41575-025-01092-6

[53] Vacca M, Celano G, Calabrese FM et al (2020) The controversial role of human gut Lachnospiraceae. Microorganisms 8:573. 10.3390/microorganisms8040573 32326636 10.3390/microorganisms8040573 PMC7232163

[54] Calderon G, McRae A, Rievaj J et al (2020) Ileo-colonic delivery of conjugated bile acids improves glucose homeostasis via colonic GLP-1-producing enteroendocrine cells in human obesity and diabetes. eBioMedicine 55:102759. 10.1016/j.ebiom.2020.102759 32344198 10.1016/j.ebiom.2020.102759 PMC7186521

[55] Barrasa JI, Olmo N, Lizarbe MA, Turnay J (2013) Bile acids in the colon, from healthy to cytotoxic molecules. Toxicol Vitro 27:964–977. 10.1016/j.tiv.2012.12.020 10.1016/j.tiv.2012.12.020 23274766

