# Supplementary material for: Distinct effects of supplementation with resistant starch and polydextrose on plasma and faecal bile acid profile and associations with gut microbiota: a randomised, controlled intervention in healthy participants
Source: Eur J Nutr. 2026 Jul 23;65(5):220. doi: 10.1007/s00394-026-04060-1 (PMC13396052; doi:10.1007/s00394-026-04060-1)
Supplement: Supplementary file 1 — Supplementary Material 1 [file 394_2026_4060_MOESM1_ESM.docx]

**Distinct effects of supplementation with resistant starch and polydextrose on plasma and faecal bile acid profile and associations with gut microbiota: a randomised, controlled intervention in healthy participants**

Jiemin Fan^1,2^, Gwenaelle Le Gall^3^, Fiona C. Malcomson^1,2^, Panayiotis Louca^1^, Lauren Beck^4^, Andrew Nelson^5^, Naomi D. Willis^1^, Iain McCallum^6^, Long Xie^1^, Arthur C. Ouwehand^7^, Julian D. Stowell^8^, Seamus B. Kelly^6^, D. Michael Bradburn^9^, Nigel J. Belshaw^3^, Ian T. Johnson^10^, Christopher, J. Stewart^4^, Michael Müller^3^, Bernard M. Corfe^1,2^ and John C. Mathers^1,2^

1 Human Nutrition and Exercise Research Centre, Centre for Healthier Lives, Population Health Sciences Institute, Newcastle University, Newcastle upon Tyne NE2 4HH, UK

2 Centre for Cancer, Population Health Sciences Institute, Newcastle University, Newcastle upon Tyne NE2 4HH, UK

3 Faculty of Medicine and Health Sciences, Norwich Medical School, University of East Anglia, Norwich, NR4 7TJ, UK

4 Translational and Clinical Research Institute, Newcastle University, Newcastle upon Tyne NE2 4HH, UK

5 Department of Applied Science, Northumbria University, Newcastle upon Tyne NE1 8ST, UK

6 Northumbria Healthcare NHS Foundation Trust, North Tyneside General Hospital, Rake Lane, North Shields NE29 8NH, UK

7 IFF Health, Kantvik 02460, Finland

8 Sabri Ülker Foundation, Istanbul, Turkey

9 Northumbria Healthcare National Health Service Foundation Trust, NE63 9JJ, Ashington, UK

10Quadram Institute, Norwich Research Park, Norwich, Norfolk NR4 7UQ, UK

**Corresponding Author:** Professor Bernard Corfe, Human Nutrition and Exercise Research Centre, Population Health Sciences Institute, Newcastle University, Newcastle upon Tyne NE2 4HH, UK, email: [Bernard.Corfe@newcastle.ac.uk](mailto:Bernard.Corfe@newcastle.ac.uk), Telephone: +44 (0) 191 208 1135

Supplementary methods of LC-MS/MS of BA profiling

Preparation of calibration samples

Stock solutions of BA standards were prepared in methanol (1mg/mL) and stored at -80°C. A plasma bile acid standard mixture (plasma STD mix) was prepared by pooling 19 bile acid standards. In order to construct calibration curves for quantification analysis, the standards mixture was further diluted to seven gradient concentrations between 0.0625 ppm and 4 ppm as calibration samples. 25µl of plasma STD mix of each calibration curve point was pipetted into glass vials. 25µl of MeOH was pipetted for the 0 ppm. 15 µl of GCDCA-d4 and 15µl of CA-d4 were added at 5 ppm. A faecal bile acid standard mixture (faecal STD mix) was prepared by pooling 25 bile acid standards. The standards mixture was then diluted to five gradient concentrations as calibration samples of each bile acid around its expected concentration range. 50µl from each gradient dilution was pipetted into glass vials. 50µl of MeOH was pipetted for the 0 ppm and 30µl of the mixture of internal standards (GCDCA-d4, CDCA-d4, DCA-d4, LCA-d4) were then added at 5 ppm each.

Quality Control (QC) sample preparation

Plasma QC samples were prepared by pooling aliquots of 20µl from all study samples[1]. Faecal QC samples were prepared by pooling approximate 500 μL faeces from all study samples[2]. All QC samples were processed with study samples using identical extraction protocol.

LC-MS/MS Condition

BA profiling was performed using ultra-performance liquid chromatography mass spectrometry (Waters Acquity UPLC system and Xevo TQ-S Cronos mass spectrometer, Waters) controlled by MassLynx 4.1 software. The electrospray ionization operated in negative mode and chromatographic separations were performed with a Supelco Ascentis Express C18 column (150 x 4.6 mm, 2.7 µM) maintained at 40 ℃. Eluent A (10mM ammonium acetate, 0.1% formic acid, water) and eluent B (10mM ammonium acetate, 0.1% formic acid, methanol) run at a constant rate of 0.6 mL/min. The gradient began at 50% B and was held for 2 min before a linear increase to 95% B occurring at 20 min. This was held for 4 min before a linear decrease in gradient back to 50% B occurred between 24 and 25 min. The gradient was held at 50%B for another 4 mins.

Analytical Performance

Calibration standards were run at the beginning and end of each experimental batch. Calibration curves for each BA were constructed by fitting standard concentrations to the ratio of standard peak area to internal standard peak area. An excellent linear response range was ensured, with correlation coefficients (r²) of 0.99 or higher for all calibration curves. An Agilent high-performance autosampler with an injection programme minimised carry-over effects between samples. 20% of study samples in each batch were reanalysed to monitor intra-batch variations.

References

1. Choucair I, Nemet I, Li L, et al (2020) Quantification of bile acids: a mass spectrometry platform for studying gut microbe connection to metabolic diseases. J Lipid Res 61:159–177. https://doi.org/10.1194/jlr.RA119000311

2. Zawadzki AD, Thiele M, Suvitaival T, et al (2022) High-throughput UHPLC-MS to screen metabolites in feces for gut metabolic health. Metabolites 12:211. https://doi.org/10.3390/metabo12030211

Supplementary Table 1 Categorisation of bile acids in the DISC Study

| Bile acids ^a^ | Primary BAs | Secondary BAs | Conjugated BAs | Keto BAs |
| --- | --- | --- | --- | --- |
| CA | **×** |  |  |  |
| GCA | **×** |  | **×** |  |
| TCA | **×** |  | **×** |  |
| CDCA | **×** |  |  |  |
| GCDCA | **×** |  | **×** |  |
| TCDCA | **×** |  | **×** |  |
| DCA |  | **×** |  |  |
| GDCA |  | **×** | **×** |  |
| TDCA |  | **×** | **×** |  |
| GLCA |  | **×** | **×** |  |
| GUDCA |  | **×** | **×** |  |
| LCA |  | **×** |  |  |
| IsoLCA |  | **×** |  |  |
| HDCA |  | **×** |  |  |
| 3-keto DCA |  |  |  | **×** |
| 3-keto LCA |  |  |  | **×** |
| 12-keto LCA |  |  |  | **×** |
| a. Abbreviations: CA, cholic acid; GCA, glycocholic acid; TCA, taurocholic acid; CDCA, chenodeoxycholic acid; GCDCA, glycochenodeoxycholic acid; TCDCA, taurochenodeoxycholic acid; DCA, deoxycholic acid; GDCA, glycodeoxycholic acid; TDCA, taurodeoxycholic acid; GLCA, glycolithocholic acid; GUDCA, glycoursodeoxycholic acid; IsoLCA: isolithocholic acid; HDCA, hyodeoxycholic acid; LCA, lithocholic acid; 3-keto LCA, 3-ketolithocholic acid; 3-keto DCA, 3-ketodeoxycholic acid;12-keto LCA, 12-ketolithocholic acid. | | | | |

| Supplementary Table 2 Statistical approach for partial correlations between BA outcomes and genus abundances in response to RS and PD supplementation | | | | | |
| --- | --- | --- | --- | --- | --- |
| Correlation type | Subpopulation | Adjusted BA outcome ^a^ | Adjusted taxa abundance | Spearman correlation analysis |  |
| Before RS  supplementation | RS group + (RS+PD) group | lm (baseline BA outcome ~ experiment batch + covariates ^b^) | lm(baseline abundance ~ covariates ^b^) | Compute pairwise spearman correlations between residuals from BA model and residuals from taxa model |  |
| After RS  supplementation |  | lm (post-intervention BA outcome ~ **PD** + experiment batch + covariates ^c^) | lm(post-intervention abundance ~ **PD +** covariates ^c^) |  |  |
| Before PD  supplementation | PD group + (RS+PD) group | betareg (baseline BA outcome ~ experiment batch + covariates ^b^) | lm(baseline abundance ~ covariates ^b^) |  |  |
| After PD  supplementation |  | betareg (post-intervention BA outcome ~ **RS** + experiment batch + covariates ^c^) | lm(post-intervention abundance ~ **RS +** covariates ^c^) |  |  |
| a. Linear regression models were used to adjust BA concentrations (log transformed) and beta regression models were used to adjust BA proportions and secondary BA ratios.  b. Baseline covariates: age, BMI, sex, smoking status, habitual intake of alcohol, energy and fibre, endoscopy procedure. c. Post-intervention covariates: age, BMI, sex, smoking status, habitual intake of alcohol, energy and fibre. | | | | |  |

Supplementary Table 3 Demographic characteristics of 74 participants in the DISC Study with paired plasma samples at baseline and post-intervention ^a^

| Characteristics | RS- vs. RS+ | | P value ^b^ | PD- vs. PD+ | | P value ^b^ |
| --- | --- | --- | --- | --- | --- | --- |
|  | RS- | RS+ |  | PD- | PD+ |  |
| Participants (n) | 40 | 34 |  | 37 | 37 |  |
| Age (years) | 53.1 (14.7) | 51.7 (8.8) | 0.611 | 50.4 (10.5) | 54.5 (13.7) | 0.155 |
| Sex (F:M) | 21:19 | 18:16 | 1.000 | 23:14 | 16:21 | 0.162 |
| BMI (kg/m^2^) | 29.7 (5.3) | 30.4 (5.4) | 0.618 | 30.3 (4.9) | 29.8 (5.7) | 0.381 |
| Ethnicity (n) |  |  | 0.417 |  |  | 0.368 |
| Caucasian | 38 | 34 |  | 36 | 36 |  |
| Black African | 1 | 0 |  | 1 | 0 |  |
| Mixed race | 1 | 0 |  | 0 | 1 |  |
| Smoking status (n) |  |  | 0.265 | 0.265 |  | 0.896 |
| Never | 24 | 14 |  | 20 | 18 |  |
| Used | 9 | 12 |  | 10 | 11 |  |
| Yes | 7 | 8 |  | 7 | 8 |  |
| Endoscopy procedure (n) |  |  | 1.000 |  |  | 0.799 |
| Colonoscopy | 12 | 10 |  | 12 | 10 |  |
| Flexible Sigmoidoscopy | 28 | 24 |  | 25 | 27 |  |
| Dietary data |  |  |  |  |  |  |
| Energy (Kcal/day) | 2568.8 (1157.1) | 2682.5 (1697.6) | 0.859 | 2593.7 (1603.2) | 2648.4 (1235.7) | 0.286 |
| Carbohydrate intake (g/day) | 316.0 (133.4) | 327.4 (213.0) | 0.460 | 313.1 (199.9) | 329.3 (144.3) | 0.215 |
| Dietary fibre intake (g/day) | 22.3 (9.9) | 23.2 (11.9) | 0.850 | 21.8 (10.6) | 23.6 (11.0) | 0.451 |
| Fat intake (g/day) | 96.5 (53.6) | 98.0 (84.1) | 0.421 | 99.6 (82.5) | 94.7 (52.7) | 0.526 |
| Protein intake (g/day) | 92.7 (44.4) | 98.6 (47.6) | 0.493 | 94.6 (46.9) | 96.2 (45.0) | 0.667 |
| a. Data are presented as mean and (standard deviation) for continuous variables including age, BMI and dietary data, and as number of participants (n) for categorical variables including sex, ethnicity, smoking status, and endoscopy procedure.  b. P values were derived from Mann-Whitney U test or independent t test for continuous variables and derived from Chi-square test for categorical variables. | | | | | | |

Supplementary Table 4 Demographic characteristics of 50 participants in the DISC Study with paired faecal samples at baseline and post-intervention ^a^

| Characteristics | RS- vs. RS+ | | P value ^b^ | PD- vs. PD+ | | P value ^b^ |
| --- | --- | --- | --- | --- | --- | --- |
|  | RS- | RS+ |  | PD- | PD+ |  |
| Participant (n) | 24 | 26 |  | 22 | 28 |  |
| Age (years) | 55.6 (14.6) | 53.1 (9.1) | 0.476 | 53.0 (10.3) | 55.4 (13.3) | 0.469 |
| Sex (F:M) | 10:14 | 13:13 | 0.759 | 10:12 | 13:15 | 1.000 |
| BMI (kg/m2) | 30.9 (5.9) | 29.6 (5.2) | 0.360 | 31.2 (4.3) | 29.5 (6.3) | 0.074 |
| Ethnicity (n) |  |  | 0.777 |  |  | 0.396 |
| Caucasian | 24 | 26 |  | 22 | 28 |  |
| Black African | 0 | 0 |  | 0 | 0 |  |
| Mixed race | 0 | 0 |  | 0 | 0 |  |
| Smoking status (n) |  |  | 0.500 |  |  | 0.807 |
| Never | 14 | 11 |  | 12 | 13 |  |
| Used | 6 | 8 |  | 6 | 8 |  |
| Yes | 4 | 7 |  | 4 | 7 |  |
| Endoscopy procedure (n) |  |  | 1.000 |  |  | 0.779 |
| Colonoscopy | 8 | 8 |  | 8 | 8 |  |
| Flexible Sigmoidoscopy | 16 | 18 |  | 14 | 20 |  |
| Dietary data |  |  |  |  |  |  |
| Energy (Kcal/day) | 2662.8 (1273.7) | 2640.6 (1627.0) | 0.623 | 2737.5 (1919.8) | 2583.5 (977.6) | 0.410 |
| Carbohydrate intake (g/day) | 332.4 (153.6) | 326.1 (209.4) | 0.446 | 339.6 (243.8) | 320.9 (119.7) | 0.399 |
| Dietary fibre intake (g/day) | 23.3 (11.4) | 23.5 (12.3) | 0.955 | 23.8 (12.4) | 23.1 (11.5) | 0.823 |
| Fat intake (g/day) | 99.6 (55.4) | 96.5 (85.7) | 0.330 | 103.9 (99.1) | 93.4 (41.5) | 0.410 |
| Protein intake (g/day) | 93.0 (45.8) | 96.8 (42.2) | 0.651 | 94.9 (50.4) | 95.0 (38.3) | 0.649 |
| a. Data are presented as mean and (standard deviation) for continuous variables including age, BMI and dietary data, and as number of participants (n) for categorical variables including sex, ethnicity, smoking status, and endoscopy procedure.  b. P values were derived from Mann-Whitney U test or independent t test for continuous variables and derived from Chi-square test for categorical variables. | | | | | | |

| Supplementary Table 5 Concentrations (nmol/L) of BAs in plasma at baseline ^a^ | | | | | | |
| --- | --- | --- | --- | --- | --- | --- |
| BAs/ BA category ^c^ | RS- vs. RS+ | | P value ^b^ | PD- vs. PD+ | | P value ^b^ |
|  | RS- (n= 40) | RS+ (n=34) |  | PD- (n=37) | PD+ (n=37) |  |
| Total BAs | 1559.47 (1449.55) | 1366.42 (1249.67) | 0.454 | 1396.37 (1177.67) | 1545.17 (1525.97) | 0.795 |
| Primary BAs | 1106.86 (1177.1) | 922.11 (950.72) | 0.345 | 945.65 (865.15) | 1098.3 (1259.58) | 0.804 |
| Secondary BAs | 452.6 (394.03) | 444.32 (360.69) | 0.841 | 450.73 (393.95) | 446.87 (363.71) | 0.974 |
| Glycine conjugated BAs | 948.34 (780.68) | 863.85 (942.59) | 0.351 | 885.01 (855.03) | 934.03 (863.72) | 0.689 |
| Taurine conjugated BAs | 108.58 (73.33) | 96.17 (86.1) | 0.225 | 101.67 (77.45) | 104.09 (81.85) | 0.879 |
| Unconjugated BAs | 502.55 (875.16) | 406.4 (621.47) | 0.849 | 409.69 (617.9) | 507.06 (895.44) | 0.721 |
| CA | 113.26 (287.26) | 90.64 (209.3) | 0.696 | 83.69 (198.48) | 122.04 (299.43) | 0.844 |
| GCA | 154.42 (161.03) | 140.48 (155.81) | 0.652 | 147.93 (168.9) | 148.11 (148.04) | 0.948 |
| TCA | 21.45 (17.21) | 21.79 (19.33) | 0.554 | 22.14 (18.17) | 21.07 (18.24) | 0.711 |
| CDCA | 236.82 (481.96) | 168.45 (331.34) | 0.678 | 170.61 (316.15) | 240.2 (502.22) | 0.817 |
| GCDCA | 518.35 (449.85) | 452.36 (557.99) | 0.224 | 466.69 (481.79) | 509.37 (523.26) | 0.681 |
| TCDCA | 62.56 (47.13) | 48.39 (44.52) | 0.093 | 54.58 (44.23) | 57.51 (48.63) | 0.983 |
| DCA | 152.46 (182.86) | 147.32 (131.44) | 0.765 | 155.38 (166.78) | 144.81 (155.56) | 0.829 |
| GDCA | 173.22 (176.62) | 179.49 (202.06) | 0.511 | 176.44 (200.95) | 175.77 (175.67) | 0.927 |
| TDCA | 24.58 (22.13) | 25.99 (29.6) | 0.356 | 24.95 (25.17) | 25.5 (26.47) | 0.858 |
| GLCA | 16.62 (15.49) | 23.12 (16.84) | 0.076 | 18.76 (16.26) | 20.45 (16.59) | 0.944 |
| GUDCA | 85.72 (103.92) | 68.4 (71.19) | 0.403 | 75.19 (83.28) | 80.33 (97.72) | 0.900 |

a. Data are presented as mean and (standard deviation).

b. Mann-Whitney U test was used to test the difference between RS- vs. RS+ and PD- vs. PD+.

c. Abbreviations: CA, cholic acid; GCA, glycocholic acid; TCA, taurocholic acid; CDCA, chenodeoxycholic acid; GCDCA, glycochenodeoxycholic acid; TCDCA, taurochenodeoxycholic acid; DCA, deoxycholic acid; GDCA, glycodeoxycholic acid; TDCA, taurodeoxycholic acid; GLCA, glycolithocholic acid; GUDCA, glycoursodeoxycholic acid.

Supplementary Table 6 Proportions (%) of BAs in plasma at baseline ^a^

| BAs/ BA category ^c^ | RS- vs. RS+ | | P value ^b^ | PD- vs. PD+ | | P value ^b^ |
| --- | --- | --- | --- | --- | --- | --- |
|  | RS- (n= 40) | RS+ (n=34) |  | PD- (n=37) | PD+ (n=37) |  |
| Primary BAs | 67.23 (10.65) | 64.04 (13.4) | 0.435 | 64.9 (11.45) | 66.63 (12.65) | 0.517 |
| Secondary BAs | 32.77 (10.65) | 35.96 (13.4) | 0.435 | 35.1 (11.45) | 33.37 (12.65) | 0.517 |
| Glycine conjugated BAs | 62.05 (15.35) | 59.07 (17.33) | 0.474 | 60.19 (16.79) | 61.17 (15.89) | 0.681 |
| Taurine conjugated BAs | 9.64 (5.32) | 9.4 (5.35) | 0.795 | 9.76 (5.46) | 9.31 (5.2) | 0.779 |
| Unconjugated BAs | 28.31 (16.93) | 31.53 (19.12) | 0.515 | 30.05 (18.38) | 29.52 (17.69) | 0.897 |
| CA | 5.17 (5.8) | 5.66 (6.12) | 0.761 | 5.16 (5.63) | 5.62 (6.25) | 0.948 |
| GCA | 9.76 (4.91) | 10.12 (5.04) | 0.931 | 10.52 (5.75) | 9.33 (3.96) | 0.559 |
| TCA | 2.13 (1.56) | 2.49 (1.88) | 0.588 | 2.43 (1.88) | 2.16 (1.53) | 0.705 |
| CDCA | 12.25 (8.94) | 12.52 (10.62) | 0.820 | 11.86 (9.27) | 12.89 (10.18) | 0.770 |
| GCDCA | 32.64 (12.37) | 28.67 (11.99) | 0.186 | 29.88 (12.26) | 31.75 (12.4) | 0.552 |
| TCDCA | 5.29 (3.08) | 4.58 (2.46) | 0.324 | 5.04 (2.76) | 4.88 (2.91) | 0.604 |
| DCA | 10.89 (7.79) | 13.35 (11.37) | 0.448 | 13.03 (9.9) | 11.01 (9.34) | 0.375 |
| GDCA | 11.81 (5.87) | 12.08 (6.61) | 0.974 | 11.76 (5.13) | 12.11 (7.14) | 0.787 |
| TDCA | 2.23 (1.55) | 2.33 (1.71) | 0.871 | 2.29 (1.54) | 2.27 (1.72) | 0.829 |
| GLCA | 2.18 (3.2) | 2.91 (2.79) | 0.018* | 2.19 (1.82) | 2.85 (3.87) | 0.922 |
| GUDCA | 5.66 (4.08) | 5.29 (4.02) | 0.948 | 5.84 (4.68) | 5.13 (3.27) | 0.729 |
| a. Data are presented as mean and (standard deviation).  b. Mann-Whitney U test was used to test the difference between RS- vs. RS+ and PD- vs. PD+. *P < 0.05 was significantly different.  c. Abbreviations: CA, cholic acid; GCA, glycocholic acid; TCA, taurocholic acid; CDCA, chenodeoxycholic acid; GCDCA, glycochenodeoxycholic acid; TCDCA, taurochenodeoxycholic acid; DCA, deoxycholic acid; GDCA, glycodeoxycholic acid; TDCA, taurodeoxycholic acid; GLCA, glycolithocholic acid; GUDCA, glycoursodeoxycholic acid. | | | | | | |

| Supplementary Table 7 Concentrations (nmol/g wet weight) of BAs in faeces at baseline ^a^ | | | | | | |
| --- | --- | --- | --- | --- | --- | --- |
| BAs/ BA category ^c^ | RS- vs. RS+ | | P value ^b^ | PD- vs. PD+ | | P value ^b^ |
|  | RS- (n=24) | RS+ (n=26) |  | PD- (n=22) | PD+ (n=28) |  |
| Total BAs | 4291.22 (2208.45) | 3075.73 (2441.55) | 0.040* | 3304.88 (2161.26) | 3937.54 (2557.63) | 0.455 |
| Primary BAs | 62.56 (153.43) | 64.58 (89.82) | 0.413 | 57.6 (96.66) | 68.33 (142.15) | 0.367 |
| Secondary BAs | 3498.77 (1905.63) | 2540.41 (2141.24) | 0.042* | 2619.18 (1762.1) | 3299.96 (2266.45) | 0.356 |
| Keto BAs | 729.9 (504.43) | 470.75 (356.1) | 0.038* | 628.1 (547.93) | 569.25 (360.72) | 0.900 |
| CA | 28.46 (80.58) | 27.88 (48.04) | 0.521 | 29.13 (59.44) | 27.4 (70.13) | 0.747 |
| CDCA | 30.38 (74.17) | 33.39 (47.47) | 0.291 | 24.81 (37.34) | 37.55 (74.96) | 0.360 |
| GCDCA | 3.72 (2.46) | 3.31 (1.42) | 0.810 | 3.66 (1.64) | 3.39 (2.23) | 0.176 |
| DCA | 1448.09 (960.87) | 1070.22 (1166.52) | 0.063 | 1119.8 (850.28) | 1355.16 (1234.86) | 0.649 |
| LCA | 1482.12 (783.81) | 1097.39 (777.2) | 0.066 | 1071.31 (726.27) | 1447.65 (822.3) | 0.116 |
| IsoLCA | 313.63 (187.51) | 205.06 (116.46) | 0.036* | 245.28 (162.86) | 266.52 (164.68) | 0.706 |
| HDCA | 254.93 (162.78) | 167.73 (173.08) | 0.022* | 182.79 (162.94) | 230.63 (179.26) | 0.290 |
| 3-keto DCA | 45.95 (35.7) | 29 (26) | 0.069 | 39.85 (36.47) | 35.01 (28.27) | 0.799 |
| 3-keto LCA | 195.18 (190.71) | 115.18 (100.67) | 0.144 | 159.96 (187.12) | 148.57 (126.62) | 0.854 |
| 12-keto LCA | 488.77 (318.14) | 326.56 (269.46) | 0.049* | 428.29 (356.1) | 385.67 (257.21) | 0.883 |

a. Data are presented as mean and (standard deviation).

b. Mann-Whitney U test was used to test the difference between RS- vs. RS+ and PD- vs. PD+. *P < 0.05 was significantly different.

c. Abbreviations: CA, cholic acid; CDCA, chenodeoxycholic acid; GCDCA, glycochenodeoxycholic acid; DCA, deoxycholic acid; IsoLCA: isolithocholic acid; HDCA, hyodeoxycholic acid; LCA, lithocholic acid; 3-keto LCA, 3-ketolithocholic acid; 3-keto DCA, 3-ketodeoxycholic acid;12-keto LCA, 12-ketolithocholic acid.

| Supplementary Table 8 Proportions (%) of BAs in faeces at baseline ^a^ | | | | | | | |
| --- | --- | --- | --- | --- | --- | --- | --- |
| BAs/ BA category ^c^ | RS- vs. RS+ | | P value ^b^ | PD- vs. PD+ | | P value ^b^ |  |
|  | RS- (n=24) | RS+ (n=26) |  | PD- (n=22) | PD+ (n=28) |  |  |
| Primary BAs | 1.91 (4.48) | 3.16 (5.64) | 0.010* | 3.5 (6.35) | 1.83 (3.82) | 0.581 |  |
| Secondary BAs | 80.76 (7.93) | 80.19 (7.82) | 0.751 | 77.8 (8.66) | 82.56 (6.46) | 0.056 |  |
| Keto BAs | 17.32 (7.49) | 16.65 (7.19) | 0.962 | 18.7 (7.82) | 15.61 (6.63) | 0.189 |  |
| CA | 0.87 (2.42) | 1.45 (3.32) | 0.014* | 1.79 (3.82) | 0.68 (1.85) | 0.594 |  |
| CDCA | 0.91 (2.11) | 1.52 (2.33) | 0.020* | 1.51 (2.5) | 1.01 (2.01) | 0.581 |  |
| GCDCA | 0.13 (0.12) | 0.19 (0.18) | 0.042* | 0.2 (0.19) | 0.13 (0.12) | 0.299 |  |
| DCA | 32.86 (9.29) | 28.25 (12.71) | 0.258 | 30.72 (12.41) | 30.26 (10.63) | 0.720 |  |
| LCA | 34.85 (7.81) | 38.94 (9.79) | 0.155 | 34.01 (8.85) | 39.31 (8.65) | 0.013* |  |
| IsoLCA | 7.31 (2.54) | 8.45 (3.97) | 0.494 | 8.34 (3.64) | 7.56 (3.18) | 0.581 |  |
| HDCA | 5.74 (1.62) | 4.55 (1.81) | 0.050 | 4.72 (1.92) | 5.43 (1.68) | 0.136 |  |
| 3-keto DCA | 4.62 (3.73) | 5.03 (4.45) | 0.825 | 5.07 (3.87) | 4.65 (4.3) | 0.594 |  |
| 3-keto LCA | 1.05 (0.54) | 0.93 (0.5) | 0.435 | 1.1 (0.54) | 0.9 (0.5) | 0.196 |  |
| 12-keto LCA | 11.65 (4.24) | 10.69 (3.92) | 0.623 | 12.53 (4.38) | 10.06 (3.51) | 0.031* |  |
| a. Data are presented as mean and (standard deviation).  b. Mann-Whitney U test was used to test the difference between RS- vs. RS+ and PD- vs. PD+. *P < 0.05 was significantly different.  c. Abbreviations: CA, cholic acid; CDCA, chenodeoxycholic acid; GCDCA, glycochenodeoxycholic acid; DCA, deoxycholic acid; IsoLCA: isolithocholic acid; HDCA, hyodeoxycholic acid; LCA, lithocholic acid; 3-keto LCA, 3-ketolithocholic acid; 3-keto DCA, 3-ketodeoxycholic acid;12-keto LCA, 12-ketolithocholic acid. | | | | | | |  |

| Supplementary Table 9 Effects of RS and PD on post-intervention proportions (%) of BAs in plasma ^a^ | | | | | | | | | |  |
| --- | --- | --- | --- | --- | --- | --- | --- | --- | --- | --- |
| BAs/ BA category ^c^ | Effect of RS | | |  | Effect of PD | | |  | P value  interaction effect |  |
|  | RS- (n = 40) | RS+ (n = 34) | P value ^b^ |  | PD- (n = 37) | PD+ (n = 37) | P value ^b^ |  |  |  |
| Primary BAs | 68.02 (2.2) | 66.38 (2.28) | 0.587 |  | 65.55 (2.24) | 68.85 (2.23) | 0.271 |  | 0.724 |  |
| Secondary BAs | 31.98 (2.2) | 33.62 (2.28) | 0.587 |  | 34.45 (2.24) | 31.15 (2.23) | 0.271 |  | 0.724 |  |
| Glycine conjugated BAs | 54.5 (2.86) | 62.64 (2.82) | 0.032* |  | 58.12 (2.83) | 59.02 (2.84) | 0.812 |  | 0.547 |  |
| Taurine conjugated BAs | 7.47 (0.68) | 7.41 (0.7) | 0.945 |  | 7.43 (0.69) | 7.45 (0.68) | 0.978 |  | 0.022* |  |
| Unconjugated BAs | 37.89 (3.11) | 28.52 (2.85) | 0.018* |  | 34.72 (2.99) | 31.7 (2.94) | 0.440 |  | 0.410 |  |
| CA | 7.14 (0.91) | 4.62 (0.7) | 0.010 |  | 6.29 (0.87) | 5.48 (0.75) | 0.416 |  | 0.570 |  |
| GCA | 8.71 (0.77) | 9.8 (0.86) | 0.297 |  | 9.38 (0.83) | 9.12 (0.81) | 0.809 |  | 0.433 |  |
| TCA | 1.62 (0.17) | 1.63 (0.17) | 0.973 |  | 1.61 (0.17) | 1.65 (0.17) | 0.861 |  | 0.147 |  |
| CDCA | 13.69 (1.66) | 10.38 (1.43) | 0.091 |  | 13.61 (1.7) | 10.47 (1.4) | 0.111 |  | 0.703 |  |
| GCDCA | 29.66 (1.7) | 31.54 (1.81) | 0.429 |  | 30.43 (1.72) | 30.77 (1.78) | 0.884 |  | 0.820 |  |
| TCDCA | 4.08 (0.36) | 3.89 (0.36) | 0.680 |  | 3.91 (0.36) | 4.05 (0.36) | 0.768 |  | 0.042 |  |
| DCA | 13.98 (1.41) | 12.31 (1.32) | 0.353 |  | 14.46 (1.42) | 11.83 (1.3) | 0.145 |  | 0.200 |  |
| GDCA | 10.45 (1.08) | 13.82 (1.38) | 0.032 |  | 12.59 (1.25) | 11.69 (1.24) | 0.576 |  | 0.223 |  |
| TDCA | 1.46 (0.17) | 1.94 (0.23) | 0.059 |  | 1.83 (0.22) | 1.57 (0.19) | 0.299 |  | 0.096 |  |
| GLCA | 1.24 (0.13) | 1.42 (0.13) | 0.288 |  | 1.48 (0.13) | 1.18 (0.12) | 0.079 |  | 0.496 |  |
| GUDCA | 3.53 (0.33) | 3.35 (0.31) | 0.658 |  | 3.47 (0.31) | 3.4 (0.32) | 0.855 |  | 0.946 |  |
| a. Data are present as estimated marginal means and (standard error) from analysing using beta regression models for the effects of RS or PD supplementation on post-intervention BA proportions. Models were adjusted for the corresponding baseline BA proportions, age, sex, BMI, smoking status, habitual intake of alcohol, energy and fibre, endoscopy procedure and experiment batches.  b. P values for the effect of RS and PD were derived from the contrast of estimated marginal means. FDR correction using Benjamini-Hochberg procedure was applied on 11 tests of proportions of individual plasma BAs. No individual BA was different between RS-vs. RS+ or between PD+ vs. PD- after FDR (all, FDR>0.05). * BA category with P < 0.05 was significantly different.  c. Abbreviations: CA, cholic acid; GCA, glycocholic acid; TCA, taurocholic acid; CDCA, chenodeoxycholic acid; GCDCA, glycochenodeoxycholic acid; TCDCA, taurochenodeoxycholic acid; DCA, deoxycholic acid; GDCA, glycodeoxycholic acid; TDCA, taurodeoxycholic acid; GLCA, glycolithocholic acid; GUDCA, glycoursodeoxycholic acid. | | | | | | | | | |  |
|  |  |  |  |  |  |  |  |  |  |  |
|  |  |  |  |  |  |  |  |  |  |  |
|  |  |  |  |  |  |  |  |  |  |  |
|  |  |  |  |  |  |  |  |  |  |  |

| Supplementary Table 10 Effects of RS and PD on post-intervention concentrations (nmol/g wet weight) of BAs in faeces ^a^ | | | | | | | | | | |
| --- | --- | --- | --- | --- | --- | --- | --- | --- | --- | --- |
| BAs/ BA category ^c^ | Effect of RS | | |  | Effect of PD | | |  | P value  interaction effect |  |
|  | RS- (n = 24) | RS+ (n = 26) | P value ^b^ |  | PD- (n = 22) | PD+ (n = 28) | P value ^b^ |  |  |  |
| Total BAs | 2394.33 (353.95) | 2585.6 (408.7) | 0.705 |  | 2753.43 (464.24) | 2248.38 (293.94) | 0.305 |  | 0.482 |  |
| Primary BAs | 29.59 (10.13) | 27 (7.56) | 0.796 |  | 24.72 (7.89) | 32.31 (9.71) | 0.439 |  | 0.533 |  |
| Secondary BAs | 1898.92 (332.5) | 2095.46 (389.68) | 0.680 |  | 2315.47 (464.54) | 1718.5 (264.89) | 0.207 |  | 0.311 |  |
| Keto BAs | 360.81 (60.13) | 458.98 (82.33) | 0.292 |  | 494.74 (92.09) | 334.73 (50.39) | 0.075 |  | 0.349 |  |
| CA | 9.47 (4.44) | 8.81 (3.41) | 0.872 |  | 7.75 (3.4) | 10.76 (4.46) | 0.453 |  | 0.524 |  |
| CDCA | 13.21 (4.51) | 13.63 (3.81) | 0.931 |  | 11.72 (3.74) | 15.36 (4.61) | 0.446 |  | 0.503 |  |
| GCDCA | 3.17 (0.17) | 2.9 (0.14) | 0.199 |  | 3.12 (0.17) | 2.95 (0.15) | 0.428 |  | 0.340 |  |
| DCA | 716.82 (179.38) | 764.11 (209.02) | 0.850 |  | 925.05 (264.53) | 592.1 (133.47) | 0.173 |  | 0.443 |  |
| LCA | 840.54 (150.01) | 966.56 (177.4) | 0.563 |  | 1046.81 (216.18) | 776.11 (120.4) | 0.229 |  | 0.265 |  |
| IsoLCA | 127.88 (32.68) | 147.08 (37.67) | 0.676 |  | 171.77 (47.84) | 109.5 (24.77) | 0.175 |  | 0.458 |  |
| HDCA | 125.49 (28.27) | 132.76 (34.11) | 0.858 |  | 159.52 (42.87) | 104.44 (20.99) | 0.165 |  | 0.502 |  |
| 3-keto DCA | 20.98 (4.73) | 21.71 (5.34) | 0.910 |  | 26.05 (6.49) | 17.48 (3.66) | 0.167 |  | 0.253 |  |
| 3-keto LCA | 67.41 (19.99) | 70.06 (21.07) | 0.920 |  | 77.75 (24.84) | 60.74 (16.28) | 0.508 |  | 0.312 |  |
| 12-keto LCA | 252 (39.86) | 339.05 (58.48) | 0.177 |  | 371.7 (65.88) | 229.86 (32.99) | 0.023 |  | 0.394 |  |
| a. Data are presented as LSMs and (standard error) from analysing using general linear models for the effects of RS or PD supplementation on post-intervention BA concentrations (log transformed). Models were adjusted for the corresponding baseline BA concentrations (log transformed), age, sex, BMI, smoking status, habitual intake of alcohol, energy and fibre, endoscopy procedure and experiment batches.  b. P values for the effect of RS and PD were derived from the contrast of LSMs. FDR correction using Benjamini-Hochberg procedure was applied on 10 tests of concentrations of individual faecal BAs.  c. Abbreviations: CA, cholic acid; CDCA, chenodeoxycholic acid; GCDCA, glycochenodeoxycholic acid; DCA, deoxycholic acid; IsoLCA: isolithocholic acid; HDCA, hyodeoxycholic acid; LCA, lithocholic acid; 3-keto LCA, 3-ketolithocholic acid; 3-keto DCA, 3-ketodeoxycholic acid;12-keto LCA, 12-ketolithocholic acid. | | | | | | | | | |  |
|  |  |  |  |  |  |  |  |  |  |  |
|  |  |  |  |  |  |  |  |  |  |  |
|  |  |  |  |  |  |  |  |  |  |  |

Supplementary Table 11 Effects of RS and PD on faecal water content (%) ^a^

| Effect of RS | | |  | Effect of PD | | |  | P value  interaction effect |
| --- | --- | --- | --- | --- | --- | --- | --- | --- |
| RS- (n = 24) | RS+ (n = 26) | P value ^b^ |  | PD- (n = 22) | PD+ (n = 28) | P value ^b^ |  |  |
| 73.6 (1.2) | 74.8(1.1) | 0.440 |  | 75.5(1.2) | 72.9(1.1) | 0.099 |  | 0.719 |
| a. Data are presented as LSMs and (standard error) from analysing using a general linear model for the effects of RS or PD supplementation on faecal water content. The model was adjusted for baseline faecal water content, age, sex, BMI, smoking status, habitual intake of alcohol, energy and fibre, endoscopy procedure.  b. P values for the effect of RS and PD were derived from the contrast of LSMs. | | | | | | | | |

| Supplementary Table 12 Partial spearman correlations between representative BA-deconjugating genus and plasma conjugated BAs in participants in response to RS supplementation ^a^ | | | | | | | | |  |
| --- | --- | --- | --- | --- | --- | --- | --- | --- | --- |
| Plasma BAs | *Bacteroides* | |  | *Bifidobacterium* | |  | *Lactobacillus* | |  |
|  | Baseline rho | Post-intervention rho | | Baseline rho | Post-intervention rho | | Baseline rho | Post-intervention rho |  |
| GCA | -0.01 | -0.03 |  | -0.19 | 0.43 |  | 0.10 | 0.35 |  |
| TCA | 0.10 | -0.07 |  | -0.13 | 0.34 |  | 0.00 | 0.41 |  |
| GCDCA | -0.20 | -0.17 |  | -0.07 | 0.39 |  | 0.26 | 0.43 |  |
| TCDCA | -0.08 | -0.10 |  | -0.09 | 0.31 |  | 0.14 | 0.36 |  |
| GDCA | -0.22 | -0.14 |  | 0.00 | 0.55 |  | 0.21 | 0.42 |  |
| TDCA | -0.08 | -0.02 |  | -0.02 | 0.52 |  | 0.12 | 0.33 |  |
| GLCA | -0.19 | 0.21 |  | -0.19 | 0.39 |  | 0.06 | -0.26 |  |
| GUDCA | -0.23 | -0.27 |  | 0.06 | 0.08 |  | 0.26 | 0.61 |  |
| Glycine conjugated BAs | -0.15 | -0.13 |  | -0.07 | 0.49 |  | 0.22 | 0.48 |  |
| Taurine conjugated BAs | -0.04 | -0.10 |  | -0.13 | 0.38 |  | 0.10 | 0.37 |  |
| a. Data are present as Spearman’s correlation coefficient from partial correlations between genus abundances and BA concentrations before (baseline rho) and after (post-intervention rho) RS supplementation in RS+ participants (n = 26). *Bacteroides* and *Bifidobacterium* were included in 45 genera that were applied with FDR correction. No significant correlations were observed after FDR correction (FDR>0.05). | | | | | | | | |  |
|  |  |  |  |  |  |  |  |  |  |
|  |  |  |  |  |  |  |  |  |  |

| Supplementary Table 13 Partial spearman correlations between *Akkermansia* and concentrations of plasma conjugated BAs in response to RS supplementation ^a^ | | | | | | | |  |
| --- | --- | --- | --- | --- | --- | --- | --- | --- |
| Plasma BAs ^c^ | Before RS supplementation | | |  | After RS supplementation | | |  |
|  | Spearman’s rho | P value | FDR ^b^ |  | Spearman’s rho | P value | FDR ^b^ |  |
| GCA | 0.18 | 0.386 | 0.724 |  | 0.47 | 0.017 | 0.682 |  |
| TCA | 0.34 | 0.094 | 0.570 |  | 0.61 | 0.001 | 0.051 |  |
| GCDCA | -0.03 | 0.894 | 0.981 |  | 0.42 | 0.034 | 0.567 |  |
| TCDCA | 0.30 | 0.135 | 0.595 |  | 0.64 | 0.001 | 0.027 |  |
| GDCA | -0.02 | 0.931 | 0.992 |  | 0.38 | 0.059 | 0.442 |  |
| TDCA | 0.23 | 0.251 | 0.870 |  | 0.49 | 0.012 | 0.255 |  |
| GLCA | -0.18 | 0.373 | 0.771 |  | 0.20 | 0.319 | 0.875 |  |
| GUDCA | -0.32 | 0.109 | 0.546 |  | -0.02 | 0.915 | 0.944 |  |
| Total BAs | -0.01 | 0.957 | 0.973 |  | 0.51 | 0.008 | 0.377 |  |
| Glycine conjugated BAs | -0.03 | 0.891 | 0.904 |  | 0.44 | 0.027 | 0.519 |  |
| Taurine conjugated BAs | 0.32 | 0.109 | 0.532 |  | 0.61 | 0.001 | 0.049 |  |
| a. Data are presented as Spearman’s correlation coefficient (Spearman’s rho) for correlations between post-intervention relative abundance of *Akkermansia* and post-intervention BA concentrations in RS+ participants (n =26).  b. FDR correction was applied on 45 genera for each BA investigated.  c. Abbreviations: GCA, glycocholic acid; TCA, taurocholic acid; GCDCA, glycochenodeoxycholic acid; TCDCA, taurochenodeoxycholic acid; GDCA, glycodeoxycholic acid; TDCA, taurodeoxycholic acid; GLCA, glycolithocholic acid; GUDCA, glycoursodeoxycholic acid. | | | | | | | |  |
|  |  |  |  |  |  |  |  |  |
|  |  |  |  |  |  |  |  |  |
|  |  |  |  |  |  |  |  |  |


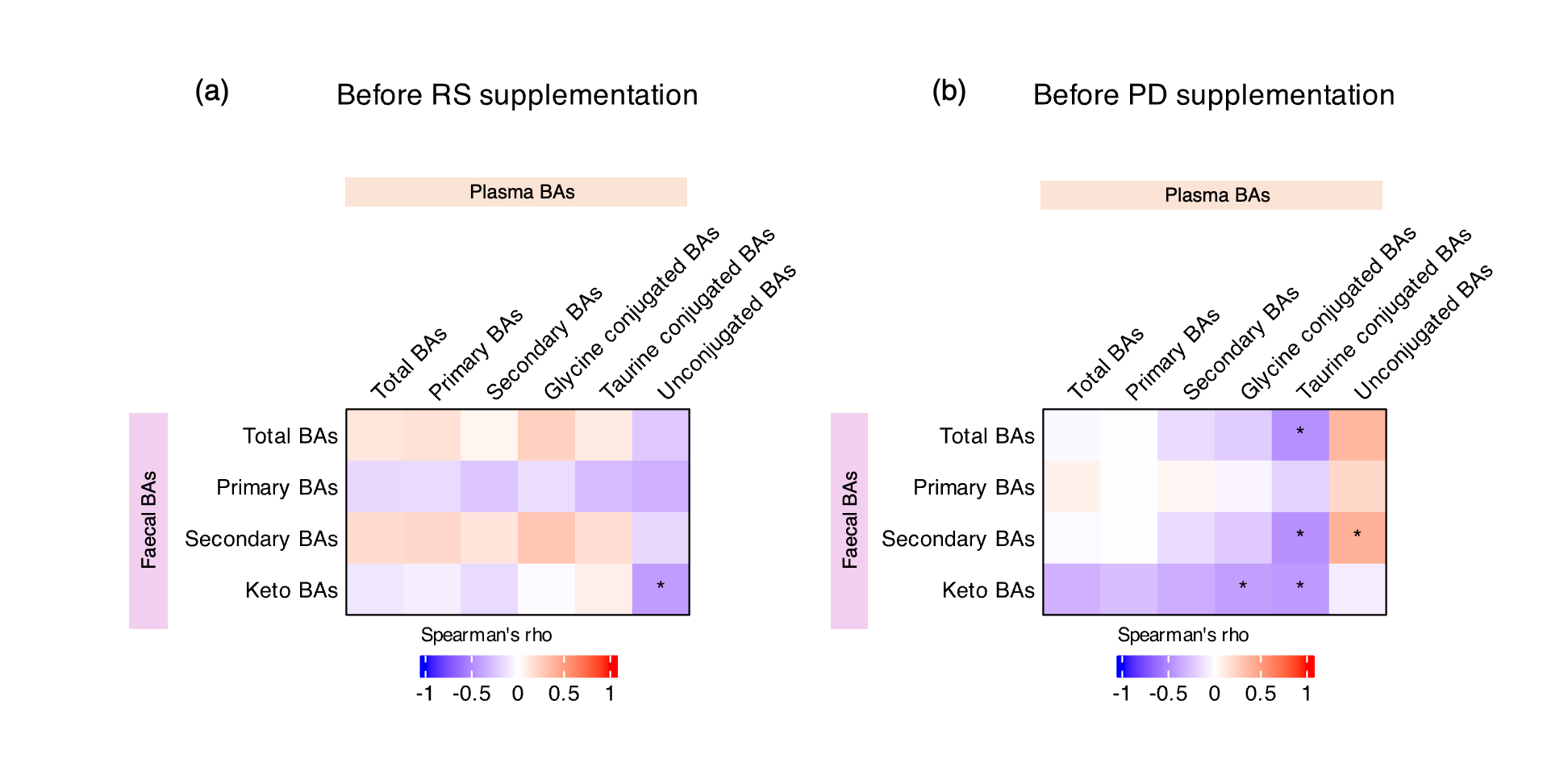


**Supplementary Fig.1** Partial spearman correlations between concentrations of BAs in plasma and faeces (a) before RS supplementation (RS+ participants, n = 25) and (b) before PD supplementation (PD+ participants, n = 27). Spearman correlations were adjusted for age, BMI, sex, smoking status, habitual intake of alcohol, energy and fibre, experimental batch and endoscopy procedure. *Unadjusted P value < 0.05.
